# A first-in-class pan-lysyl oxidase inhibitor impairs stromal remodeling and enhances gemcitabine response and survival in pancreatic cancer

**DOI:** 10.1038/s43018-023-00614-y

**Published:** 2023-08-28

**Authors:** Jessica L. Chitty, Michelle Yam, Lara Perryman, Amelia L. Parker, Joanna N. Skhinas, Yordanos F. I. Setargew, Ellie T. Y. Mok, Emmi Tran, Rhiannon D. Grant, Sharissa L. Latham, Brooke A. Pereira, Shona C. Ritchie, Kendelle J. Murphy, Michael Trpceski, Alison D. Findlay, Pauline Melenec, Elysse C. Filipe, Audrey Nadalini, Sipiththa Velayuthar, Gretel Major, Kaitlin Wyllie, Michael Papanicolaou, Shivanjali Ratnaseelan, Phoebe A. Phillips, George Sharbeen, Janet Youkhana, Alice Russo, Antonia Blackwell, Jordan F. Hastings, Morghan C. Lucas, Cecilia R. Chambers, Daniel A. Reed, Janett Stoehr, Claire Vennin, Ruth Pidsley, Anaiis Zaratzian, Andrew M. Da Silva, Michael Tayao, Brett Charlton, David Herrmann, Max Nobis, Susan J. Clark, Andrew V. Biankin, Amber L. Johns, David R. Croucher, Adnan Nagrial, Anthony J. Gill, Sean M. Grimmond, Australian Pancreatic Cancer Genome Initiative (APGI), Lorraine A. Chantrill, Angela Chou, Tanya Dwarte, Xanthe L. Metcalf, Gloria Jeong, Lara Kenyon, Nicola Waddell, John V. Pearson, Ann-Marie Patch, Katia Nones, Felicity Newell, Pamela Mukhopadhyay, Venkateswar Addala, Stephen Kazakoff, Oliver Holmes, Conrad Leonard, Scott Wood, Oliver Hofmann, Jaswinder S. Samra, Nick Pavlakis, Jennifer Arena, Hilda A. High, Ray Asghari, Neil D. Merrett, Amitabha Das, Peter H. Cosman, Kasim Ismail, Alina Stoita, David Williams, Allan Spigellman, Duncan McLeo, Judy Kirk, James G. Kench, Peter Grimison, Charbel Sandroussi, Annabel Goodwin, R. Scott Mead, Katherine Tucker, Lesley Andrews, Michael Texler, Cindy Forrest, Mo Ballal, David Fletcher, Maria Beilin, Kynan Feeney, Krishna Epari, Sanjay Mukhedkar, Nikolajs Zeps, Nan Q. Nguyen, Andrew R. Ruszkiewicz, Chris Worthley, John Chen, Mark E. Brooke-Smith, Virginia Papangelis, Andrew D. Clouston, Andrew P. Barbour, Thomas J. O’Rourke, Jonathan W. Fawcett, Kellee Slater, Michael Hatzifotis, Peter Hodgkinson, Mehrdad Nikfarjam, James R. Eshleman, Ralph H. Hruban, Christopher L. Wolfgang, Aldo Scarpa, Rita T. Lawlor, Vincenzo Corbo, Claudio Bassi, Nigel B. Jamieson, David K. Chang, Stephan B. Dreyer, Australian Pancreatic Cancer Matrix Atlas (APMA), Lea Abdulkhalek, Tatjana Schmitz, Victoria Lee, Kym Pham Stewart, Mehreen Arshi, Angela M. Steinmann, Marina Pajic, Paul Timpson, Wolfgang Jarolimek, Thomas R. Cox

**Affiliations:** 1 Cancer Ecosystems Program, The Garvan Institute of Medical Research and The Kinghorn Cancer Centre, Darlinghurst, New South Wales Australia; 2 School of Clinical Medicine, St Vincent’s Healthcare Clinical Campus, UNSW Medicine and Health, UNSW Sydney, Sydney, New South Wales Australia; 3 Pharmaxis, Frenchs Forest, New South Wales Australia; 4 The Garvan Institute of Medical Research and The Kinghorn Cancer Centre, Darlinghurst, New South Wales Australia; 5 School of Biomedical Sciences, Faculty of Medicine, Lowy Cancer Research Centre, UNSW Sydney, Sydney, New South Wales Australia; 6 Wolfson Wohl Cancer Research Centre, School of Cancer Sciences, University of Glasgow, Glasgow, UK; 7 West of Scotland Pancreatic Unit, Glasgow Royal Infirmary, Glasgow, UK; 8 Department of Medical Oncology, Westmead Hospital, Sydney, New South Wales Australia; 9 Sydney Medical School, University of Sydney, Sydney, New South Wales Australia; 10 NSW Health Pathology, Department of Anatomical Pathology, Royal North Shore Hospital, Sydney, New South Wales Australia; 11 Cancer Diagnosis and Pathology Research Group, Kolling Institute of Medical Research, Sydney, New South Wales Australia; 12 University of Melbourne Centre for Cancer Research, VCCC, Melbourne, Victoria Australia; 13 Wollongong Hospital, Illawarra and Shoalhaven Local Health District, Wollongong, New South Wales Australia; 14 Royal North Shore Hospital, St Leonards, New South Wales Australia; 15 QIMR Berghofer Medical Research Institute, Herston, Queensland Australia; 16 Bankstown Hospital, Bankstown, New South Wales Australia; 17 Liverpool Hospital, Liverpool, New South Wales Australia; 18 St Vincent’s Hospital, Darlinghurst, New South Wales Australia; 19 Westmead Hospital, Westmead, New South Wales Australia; 20 Royal Prince Alfred Hospital, Camperdown, New South Wales Australia; 21 Prince of Wales Hospital, Randwick, New South Wales Australia; 22 Fremantle Hospital, Fremantle, Western Australia Australia; 23 St John of God Healthcare, Subiaco, Western Australia Australia; 24 Epworth HealthCare, Richmond, Victoria Australia; 25 Royal Adelaide Hospital, Adelaide, South Australia Australia; 26 Flinders Medical Centre, Bedford Park, South Australia Australia; 27 Envoi Pathology, Herston, Queensland Australia; 28 Princess Alexandra Hospital, Woolloongabba, Queensland Australia; 29 Austin Hospital, Heidelberg, Victoria Australia; 30 Johns Hopkins Medical Institute, Baltimore, MD USA; 31 ARC-NET Center for Applied Research on Cancer, University of Verona, Verona, Italy; 32 University of Melbourne Centre for Cancer Research, Victorian Comprehensive Cancer Centre, Melbourne, Victoria Australia; 33 Present Address: Intravital Imaging Expertise Center, VIB Center for Cancer Biology, VIB, Leuven, Belgium

**Keywords:** Cancer, Cancer therapy

## Abstract

The lysyl oxidase family represents a promising target in stromal targeting of solid tumors due to the importance of this family in crosslinking and stabilizing fibrillar collagens and its known role in tumor desmoplasia. Using small-molecule drug-design approaches, we generated and validated PXS-5505, a first-in-class highly selective and potent pan-lysyl oxidase inhibitor. We demonstrate in vitro and in vivo that pan-lysyl oxidase inhibition decreases chemotherapy-induced pancreatic tumor desmoplasia and stiffness, reduces cancer cell invasion and metastasis, improves tumor perfusion and enhances the efficacy of chemotherapy in the autochthonous genetically engineered KPC model, while also demonstrating antifibrotic effects in human patient-derived xenograft models of pancreatic cancer. PXS-5505 is orally bioavailable, safe and effective at inhibiting lysyl oxidase activity in tissues. Our findings present the rationale for progression of a pan-lysyl oxidase inhibitor aimed at eliciting a reduction in stromal matrix to potentiate chemotherapy in pancreatic ductal adenocarcinoma.

## Abstract (web summary)

Cox and colleagues develop PXS-5505, a first-in-class selective pan-lysyl oxidase inhibitor and show that it reduces chemotherapy-induced desmoplasia and stiffness, thereby improving chemotherapy response and survival in pancreatic cancer models.

## Main

Tumor desmoplasia is a salient feature of many solid tumors and in particular pancreatic ductal adenocarcinoma (PDAC)^1^. Pancreatic cancer is known for its marked resistance to a number of therapies, including chemotherapies, radiotherapy and immunotherapy^2^. Activation of tumor-associated stromal cells and increased deposition of the extracellular matrix in the tumor microenvironment is frequently associated with the aggressive nature of PDAC^3,4^. Furthermore, there is growing recognition that many cancer therapies lead to exacerbation of fibrotic responses within tumors, further compounding these effects^5^.

Antistromal therapies that target or blunt the development of tumor desmoplasia are an emerging area with a substantial and immediate translational impact for enhancing therapy efficacy and improving survival^6–8^; however, indiscriminate ablation of the matrix, or matrix-producing cells, has yielded paradoxical results, accelerating tumor progression in in vivo models of pancreatic cancer^9,10^. Thus, a more nuanced approach, focused on stromal normalization, is likely to generate more-promising results^8,11^.

The lysyl oxidase family is critical to the biogenesis of fibrillar collagens through catalyzing the oxidative deamination of lysine residues in tropocollagen monomers thereby stabilizing them into fibrils and fibers. In mammals, there are five family homologs: lysyl oxidase (LOX) and lysyl oxidase-like 1 to 4 (LOXL1–LOXL4)^12^. Each family member shares a conserved catalytic C-terminal domain critical to their crosslinking activity, with tissue-specific expression patterns thought to play important roles in determining their exact biological function (Supplementary Table 1).

The lysyl oxidase family exhibits aberrant gene and protein expression in a number of solid tumors and its activity is closely associated with the development of tumor desmoplasia and, as a result, the family has emerged as a potential antistromal target in cancer^13^.

Ongoing efforts to develop small-molecule compounds targeting single members of the lysyl oxidase family have primarily focused on fibrotic diseases (reviewed previously^14^). Small-molecule and function-blocking antibody approaches against single family members, LOX^15,16^ and LOXL2 (refs. ^17,18^) have been used with some success in various in vitro and in vivo cancer models^17,19^ yet have yielded limited success during translation into phase 2 clinical trials, likely a result of the critical involvement of other lysyl oxidase family members, suggesting that a pan-lysyl oxidase inhibitor may be more therapeutically effective.

Herein we report the development of PXS-5505, a first-in-class small-molecule selective mechanistic inhibitor of the entire lysyl oxidase family^20^. PXS-5505 mechanistically and irreversibly inhibits all members of the lysyl oxidase family with low micromolar potency, with recovery of lysyl oxidase family activity possible only through de novo synthesis of enzyme(s).

We demonstrate efficacy of PXS-5505 in in vitro three-dimensional (3D) organotypic models of pancreatic cancer and in blocking human and mouse cancer-associated fibroblast (CAF)-driven remodeling and crosslinking of fibrillar collagen matrices, with functional effects on tissue mechanics and pancreatic cancer cell invasion. We show that daily administration of PXS-5505 in a genetically engineered mouse model and human patient-derived xenograft (PDX) models of PDAC, leads to a decrease in tumor desmoplasia, reduces tumor stiffness, improves tumor perfusion and potentiates the therapeutic efficacy of gemcitabine chemotherapy. PXS-5505 in combination with gemcitabine also decreases overt metastatic colonization of the liver at early stages and shows efficacy against established secondary liver metastases.

PXS-5505 has an excellent safety, pharmacokinetics and pharmacodynamic profile and shows complete target engagement thereby de-risking future clinical development. Together these data highlight the promise of combining antistromal therapies with already approved cancer therapies and warrant further clinical trials of PXS-5505.

## Results

### The lysyl oxidase family predicts for poor outcome in PDAC

**Extended Data Fig. 1:**
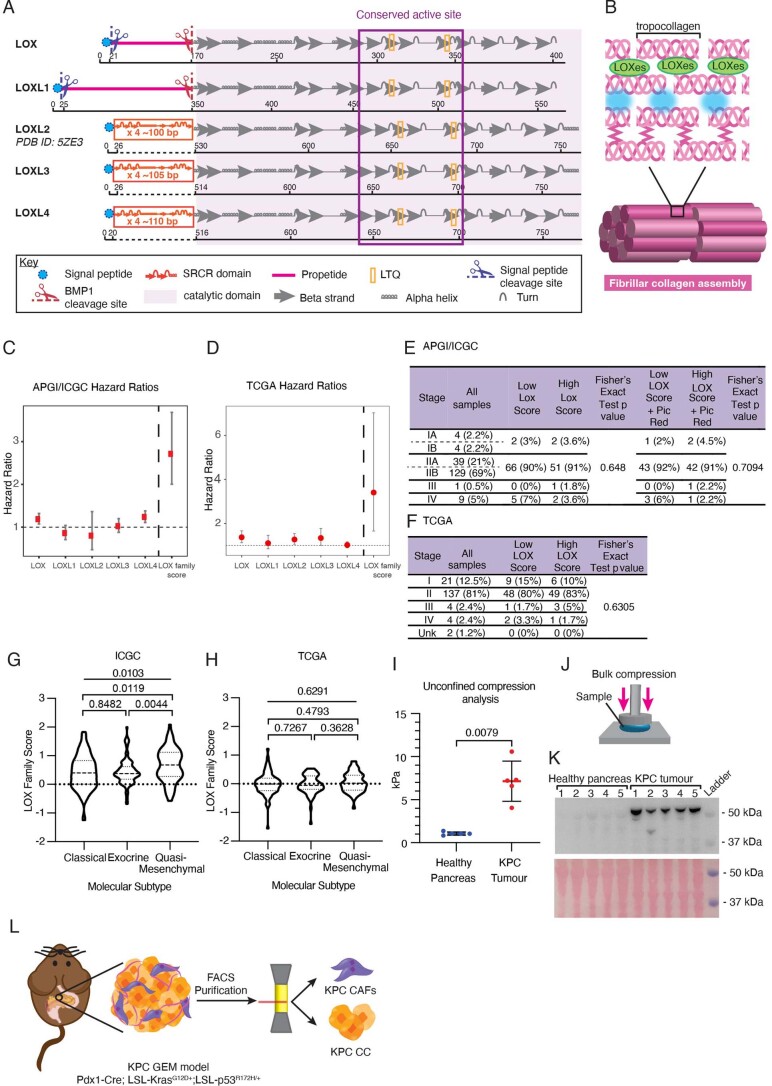
The role of the LOX family in PDAC. **a**. Prediction of LOX, LOXL1, 3 and 4 secondary structures based on the previously published secondary structure of LOXL2 (PDB ID: 5ZE3). Each family member contains a highly conserved catalytic domain critical to the formation of the active enzymatic site. LOXL2-4 contain four repeat scavenger receptor cysteine-rich (SRCR) domains (not identical sequences). LOX and LOXL1 contain additional cleavage sites and propeptide domains. **b**. Lysyl oxidases catalyze the oxidative deamination of lysine residues in the telopeptide regions of tropocollagen molecules resulting in the formation of reactive aldehydes which undergo spontaneous (blue) condensation to yield crosslinks. Further assembly of these tropocollagen molecules results in fibrillar collagen. **c, d**. Forest plots for lysyl oxidase family members showing hazard ratios for each individual family member and combined ‘lysyl oxidase family’ scores in the APGI/ICGC n = 269 patients (C) and TCGA n = 178 patients (D) cohorts. Cox proportional hazards model; dots indicate the hazard ratio and whiskers the 95% confidence interval. **e, f**. Distribution of lysyl oxidase family scores according to tumor stage in the APGI/ICGC (E) and TCGA (F) cohorts. **g, h**. Patient stratification by molecular subtype according to lysyl oxidase family score in the APGI/ICGC (G) and TGCA (H) cohorts. p value determined by Kruskal-Wallis (Nonparametric One-Way ANOVA) and unpaired, nonparametric t-test with a Mann–Whitney U correction (comparison between two groups) **i**. Quantification of bulk modulus (stiffness) by unconfined compression testing in age-matched healthy pancreas and KPC PDAC tumors (n = 5 animals per group), p value determined by unpaired, nonparametric t-test with a Mann–Whitney U correction (comparison between two groups). Data presented as mean values +/− SD. **j**. Schematic of bulk compression testing on cross-sectional slices of samples **k**. Immunoblot analysis of LOX from corresponding age-matched healthy pancreas and PDAC tumors (n = 5 animals per group) and corresponding ponceau stain as loading control. **l**. CAFs and CCs derived from the Pdx1-Cre; LSL-Kras^G12D/+^; LSL-p53^R172H/+^ (KPC) model (sorted by FACs) as previously described^46^. Source data

All lysyl oxidase family members share a conserved catalytic domain (Extended Data Fig. 1a), suggesting a similar function in collagen crosslinking (Extended Data Fig. 1b) and thus may influence PDAC progression. Analysis of the transcriptome of primary tumors from a cohort of 269 patients with PDAC from the Australian Pancreatic Cancer Genome Initiative/International Cancer Genome Consortium (APGI/ICGC) with comprehensive follow-up^21^, showed varying expression of lysyl oxidase family members in pancreatic tumor tissues. Cox proportional hazards modeling indicates that LOX and LOXL4 expression in particular, are significantly associated individually with poor overall survival in this cohort (LOX, hazard ratio (HR) 1.19 (1.07–1.319), *P* = 0.00085; LOXL4, HR 1.24 (1.108–1.379), *P* = 0.000144) (Extended Data Fig. 1c). The association of lysyl oxidase family member expression with survival was also confirmed in 178 patients from The Cancer Genome Atlas (TCGA) cohort. Here, LOX, LOXL2 and LOXL3 were significantly correlated with poor overall survival (Extended Data Fig. 1d) (LOX, HR 1.375 (1.129–1.672), *P* = 0.000594; LOXL2, HR 1.281 (1.06–1.55), *P* = 0.0101; LOXL3, HR 1.34 (1.01–1.78), *P* = 0.0408). To capture the combined contribution of all lysyl oxidase family members to survival in patients with PDAC, a ‘lysyl oxidase family score’ was calculated for each patient by weighting the individual gene expression values of each family member by their univariate Cox proportional hazards model coefficient (Extended Data Fig. 1c,d).

**Fig. 1:**
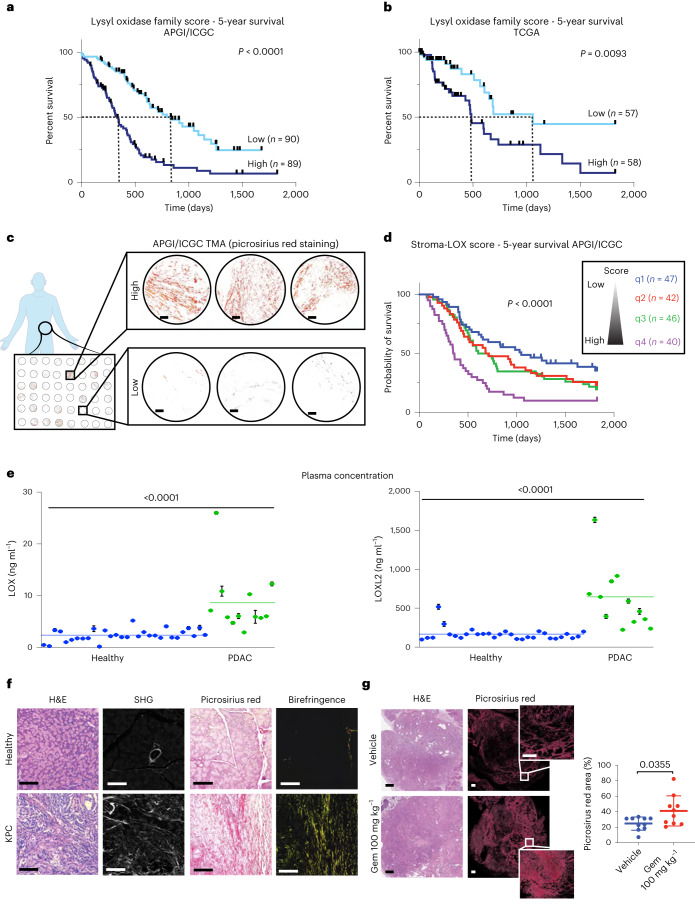
Characterization of LOX family in PDAC. **a**,**b**, Kaplan–Meier curves for 5-year overall survival for lysyl oxidase family scores in the APGI/ICGC (low, *n* = 90 patients; high, *n* = 89 patients) (**a**) and TCGA (low, *n* = 57 patients; high, *n* = 58 patients) (**b**) PDAC datasets. Lines represent upper and lower tertiles. The lysyl oxidase family expression score is weighted by the Cox proportional hazards model coefficients for each family member. *P* values determined by log-rank (Mantel–Cox) test. **c**, Representative images of picrosirius red stained tumor cores from APGI/ICGC tumor microarrays. Scale bar, 100 μm. **d**, Lysyl oxidase family score was integrated with the picrosirius red score to create a combined ‘stroma-lysyl oxidase family’ score (representing tumor fibrosis and lysyl oxidase family expression) and Kaplan–Meier curves for 5-year overall survival plotted. *P* value refers to q1 versus q4. *P* values determined by log-rank (Mantel–Cox) test. **e**, Human LOX and LOXL2 plasma concentrations in healthy (*n* = 30 patient samples) and patients with PDAC (*n* = 12 patient samples) determined by SiMoA. Data are presented as mean values and error bars represent %CV of three technical replicates from each patient. Two-tailed *P* value determined by unpaired, nonparametric *t*-test with a Mann–Whitney *U*-test correction (comparison between two groups). **f**, Representative images for comparison of age-matched healthy pancreas (taken from one of five biologically independent animals) and KPC PDAC tumor (taken from one of eighteen biologically independent animals) stained for H&E (left), imaged by multiphoton SHG imaging for collagen I (center left), picrosirius red staining viewed by transmitted light (center-right) and by polarized light (right). Scale bars, 100 μm. **g**, Quantitative comparison of picrosirius red staining in tumors from KPC tumor-bearing mice treated with either saline vehicle or gemcitabine (*n* = 10 biologically independent animals per group). Data presented as mean values ± s.d. Scale bars, 500 μm. Two-tailed *P* values determined by unpaired, nonparametric *t*-tests with a Mann–Whitney *U*-test correction (comparison between two groups). Source data

A high lysyl oxidase family score was significantly associated with worse 5-year survival in both the APGI/ICGC and TCGA cohorts (TCGA, HR 3.41 (1.658–7.023), *P* < 0.001; APGI/ICGC, HR 2.72 (2.005–3.68), *P* < 0.001) (Fig. 1a,b and Extended Data Fig. 1c,d). Our data show that the combined contribution of multiple lysyl oxidase family members is more predictive of poor survival in pancreatic cancer, than any single family member. Notably, our lysyl oxidase family score is independent of tumor stage and size. A higher lysyl oxidase score was weakly correlated with the quasi-mesenchymal molecular subtype, but only in the ICGC cohort (Extended Data Fig. 1e–h).

To understand the relationship between lysyl oxidase family expression, fibrotic collagen remodeling in the tumor microenvironment (a functional readout of lysyl oxidase family activity) and patient outcome, a patient tissue microarray containing biopsies matching the transcriptomic data from the APGI/ICGC cohort was stained for picrosirius red (Fig. 1c). Quantification of polarized light birefringent signal was integrated with our lysyl oxidase family expression score to generate a combined ‘stroma-lysyl oxidase family’ score. A low ‘stroma-lysyl oxidase family’ score, (quartile q1) consisted of tumors with low picrosirius red score and a low lysyl oxidase family score (blue). A high ‘stroma-lysyl oxidase family’ score, (quartile q4) referred to tumors with high picrosirius red score and a high lysyl oxidase family score (purple) (Fig. 1d). These data show a significant association of highly fibrotic, high lysyl oxidase family expressing tumors with poor survival (top quartile versus bottom quartile; HR 2.65 (1.659–4.29), *P* < 0.001) (Fig. 1d). In this group (q4) the median survival was 354 d compared to 1,048 d for patients with tumors that have low levels of fibrosis and low lysyl oxidase family expression (q1). These data indicate that both the level of tumor fibrosis at time of diagnosis and lysyl oxidase family expression (an indicator of ongoing/future desmoplasia) are notable determinants of patient outcome.

To further assess the link between high lysyl oxidase family expression and pancreatic cancer, we analyzed biobanked plasma from 12 patients with histologically confirmed PDAC (stage III and IV) and 30 age- and sex-matched healthy individuals with no clinical diagnosis of disease, for LOX and LOXL2. LOX and LOXL2 represent the most well-studied members of the lysyl oxidase family and a single-molecule array (SiMoA)-based detection assay^22^ was developed similar to that described previously^23^ to accurately determine their concentrations in human plasma. Our data confirm that there are significantly elevated plasma concentrations of both LOX and LOXL2 in patients with PDAC compared to healthy individuals (Fig. 1e).

### The lysyl oxidase family underpins tumor desmoplasia

The KPC mouse model of PDAC recapitulates the formation of invasive and metastatic PDAC and captures the pathology and progression of the human disease, including progressive desmoplasia^16,24^. Compared to age-matched healthy pancreata, KPC tumors show elevated stromal content, made up of high levels of fibrillar densely bundled collagens as determined by picrosirius red polarized light birefringence analysis and second harmonic generation (SHG) multiphoton imaging (Fig. 1f). We found that KPC PDAC tumors are significantly stiffer than healthy tissue (Extended Data Fig. 1i) when measured by unconfined compression analysis (Extended Data Fig. 1j) and show increased proLOX expression (Extended Data Fig. 1k).

Many first-line cancer therapies result in the activation of a fibrotic wound healing response, leading to the generation of fibrosis in and around the tumor^3^, resulting in accelerated tumor progression, promotion of resistance and relapse, and increased metastatic dissemination^25,26^. Treatment of tumor-bearing KPC mice with the chemotherapy gemcitabine (100 mg kg^−1^ or 0.9% saline control, twice weekly intraperitoneally (i.p.) for 15 cycles) led to increased deposition of fibrillar collagens compared to untreated tumors (Fig. 1g), confirming the chemotherapy mediated exacerbation of tumor desmoplasia in this clinically relevant model.

**Extended Data Fig. 2:**
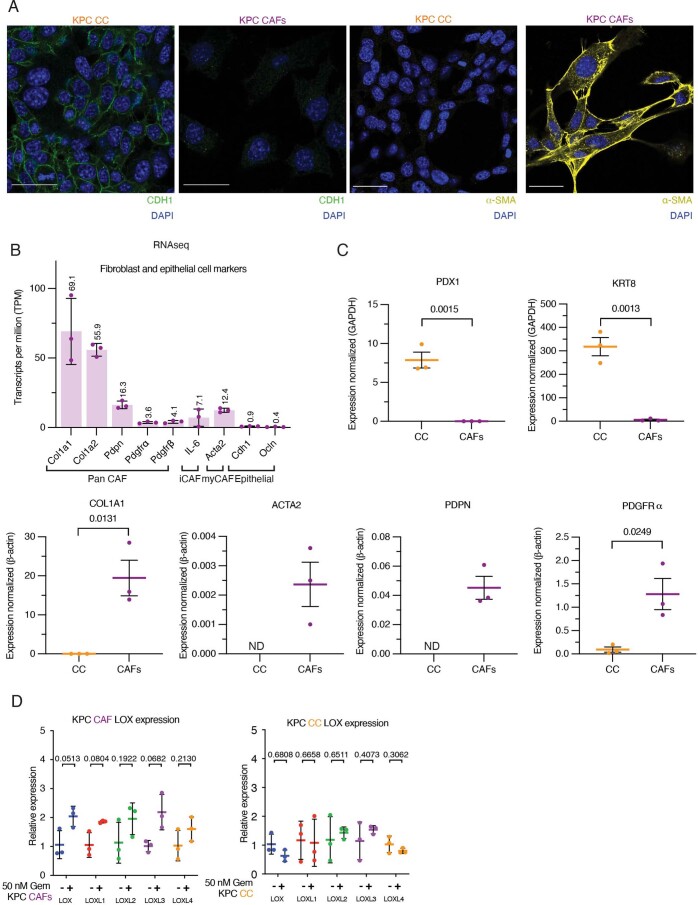
Characterization of cells derived from the KPC autochthonous model. **a**. Immunofluorescence staining of KPC CCs and KPC CAFs for the pancreatic epithelial cell marker CDH1 (green), DAPI (blue) and for the CAF marker α-SMA (yellow), DAPI (blue). Representative images from n = 3 biological independent samples. Scale bar = 30 μm. **b**. RNAseq reads for CAF and epithelial cell makers in KPC CAFs n = 3 biological independent samples. Data presented as mean values +/− SD **c**. qRT-PCR of select pancreatic epithelial cell markers and CAF markers in KPC CCs and KPC CAFs, n = 3 biological repeats with 3 technical replicates per biological repeat. p value determined by unpaired, parametric t-test. Data is presented as mean values ± SEM. (ND = not detected) **d**. qRT-PCR for lysyl oxidase family members in KPC CAFs and KPC CCs treated with 50 nM gemcitabine or vehicle control for 48 hours, n = 3 biological repeats with 3 technical replicates per biological repeat. Data presented as mean values +/− SD. Relative fold expression of LOX family members from gemcitabine treated samples compared to control were determined by 2^–∆∆Ct^ approach. *p*-values were determined by unpaired, nonparametric t-test with a Mann–Whitney U correction (comparison between two groups). Source data

CAFs are known to be the major architects of tumor desmoplasia^4,27^. To dissect the role of CAFs in PDAC desmoplasia, we used CAFs isolated from the KPC model^28^ (Extended Data Fig. 1l). We confirmed the identity of these CAFs and found them to be a ‘mixed CAF’ population, expressing a range of panCAF, myCAF and iCAF markers (Extended Data Fig. 2a–c). We also confirmed that these KPC CAFs express all lysyl oxidase family members (Extended Data Fig. 2d), which were increased upon gemcitabine administration indicating the activation of a more fibrotic CAF phenotype. Importantly, this response to gemcitabine was not observed in the matched KPC cancer cells isolated from the same model (Extended Data Fig. 2d), confirming that the CAFs are the major source of lysyl oxidase family secretion in tumors.

### PXS-5505 as a first-in-class selective and irreversible inhibitor

**Extended Data Fig. 3:**
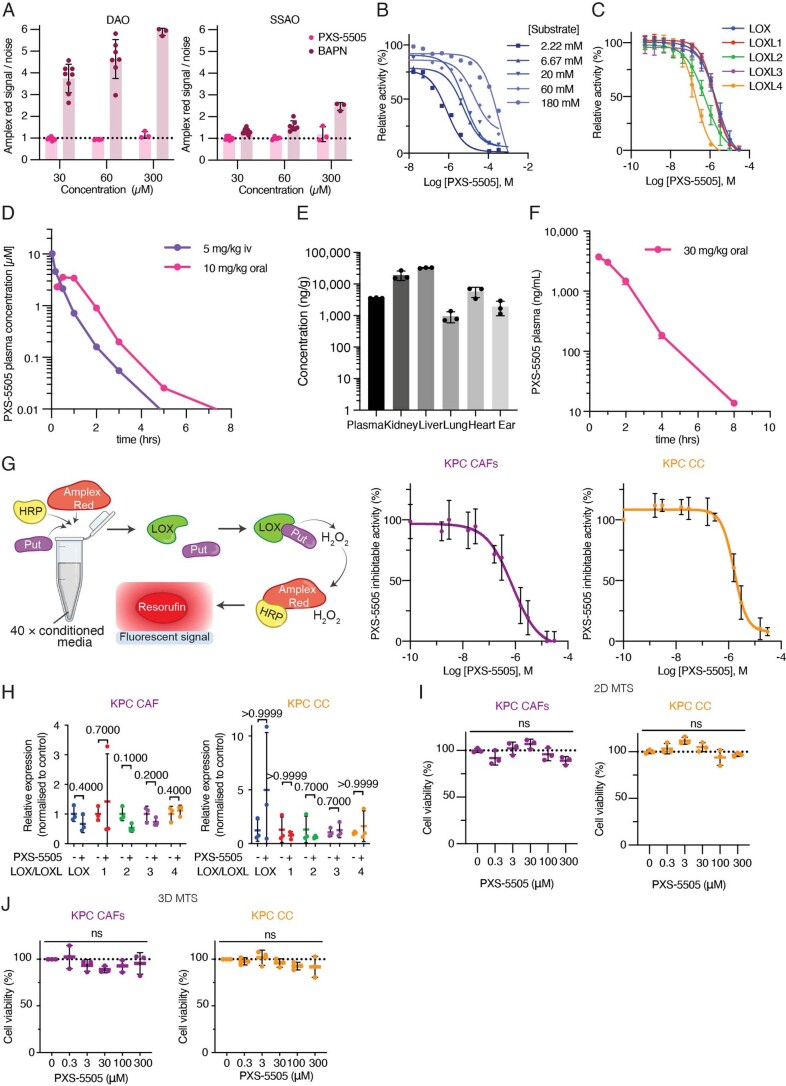
PXS-5505 is a LOX family specific inhibitor. **a**. Turnover of ßAPN by DAO (n = 8 (30 μM), 7 (60 μM), 3 (300 μM) and SSAO (n = 9 (30 μM), 7 (60 μM), 3 (300 μM) as measured by H_2_O_2_ release using an amplex red coupled fluorometric activity assay. Data presented as mean values +/− SD **b**. Substrate competition assay whereby PXS-5505 concentrations *versus* putrescine dihydrochloride is performed in an amplex red assay using bovine aorta extracted LOX (n = 3). **c**. Determination of IC_50_ of each lysyl oxidase family member by PXS-5505 n = 20 (LOX), 3 (LOXL1), 10 (LOXL2), 3 (LOXL3), 4 (LOXL4). Data presented as mean values +/− SD. **d**. Plasma concentration profile over time in rats following administration of a single dose of PXS-5505 orally (10 mg/kg) or intravenously (5 mg/kg) **e**. PXS-5505 concentration, as measured by LC–MS/MS, in rat plasma, kidney, liver, lung, heart and ear samples following a single oral dose of 30 mg/kg (n = 3 independent animals). Data presented as mean values +/− SD. **f**. Plasma concentration profile over time in mice following oral administration of PXS-5505 (30 mg/kg) (n = 3 independent animals) **g**. Schematic of amplex red coupled fluorometric lysyl oxidase family activity assay whereby putrescine (put) is used as a substrate resulting in the release of H_2_O_2_ and its subsequent catalysis by horseradish peroxidase (HRP) to reduce amplex red to resorufin resulting in a fluorescently detectable signal. Using this assay, the concentration dependent PXS-5505 inhibition of lysyl oxidase family activity in conditioned media (CM) from KPC CAFs and cancer cells was measured (n = 3 biological repeats with 3 technical replicates per biological repeat). Data presented as mean values +/− SD. **h**. qRT-PCR showing *in vitro* 48 hour treatment of KPC CAFs or cancer cells with 30 μM PXS-5505 or vehicle control (n = 3 biological repeats with 3 technical replicates per biological repeat). Data presented as mean values +/− SD. Relative fold expression of lysyl oxidase family members from PXS-5505 treated samples compared to control were determined by the 2^–∆∆Ct^ approach. *p*-values determined by unpaired, nonparametric t-test with a Mann–Whitney U correction (comparison between two groups). **i**. 2D *in vitro* cell viability assay (MTS assay) for KPC CAFs and CCs (n = 3 biological repeats with 3 technical replicates per biological repeat) in response to PXS-5505. Data presented as mean values +/− SD. Ordinary one-way ANOVA for multiple comparison test performed. **j**. 3D *in vitro* cell viability assay (MTS assay) for KPC CAFs and CCs (n = 3 biological repeats with 3 technical replicates per biological repeat) in response to PXS-5505. Data presented as mean values +/− SD. Ordinary one-way ANOVA for multiple comparison test performed. Source data

The precedence for a mechanism-based, pan-lysyl oxidase inhibitor using a small molecule has been set by the clinically tested compound β-aminopropionitrile (BAPN)^29^; however, BAPN is a small primary amine lacking moieties for selectivity and is hence a substrate for several other enzymes^30^. Specifically, BAPN is oxidized by diamine oxidase (DAO) and semicarbazide-sensitive amine oxidase (SSAO)/vascular adhesion protein 1 (VAP-1) in the vasculature (Extended Data Fig. 3a), which releases hydrogen peroxidase (H_2_O_2_) and aldehyde in humans leading to toxicity^29^.

**Fig. 2:**
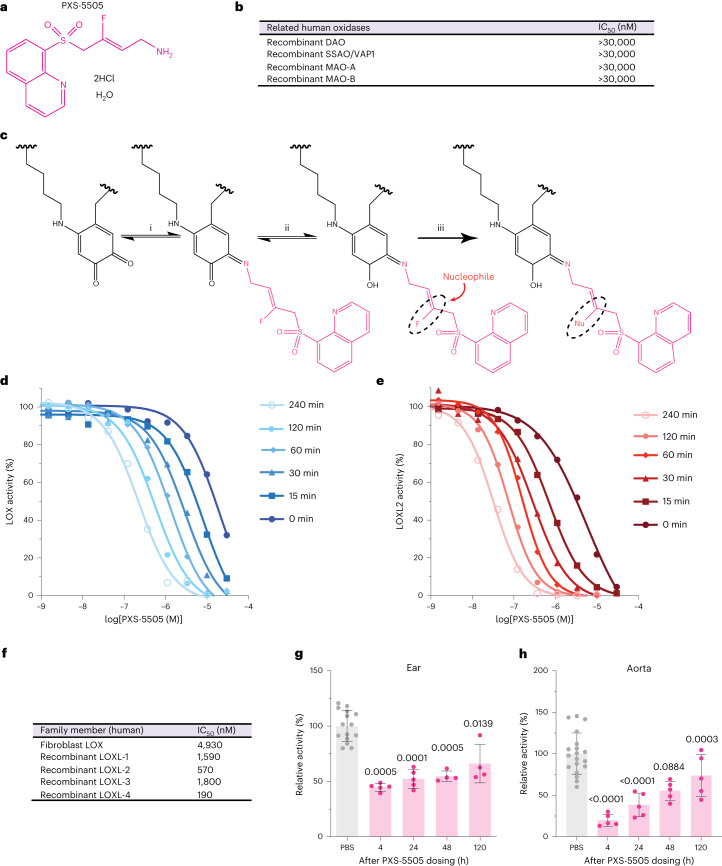
PXS-5505 is a first-in-class pan-LOX inhibitor. **a**, Chemical structure of PXS-5505. **b**, IC_50_ determination of PXS-5505 against other human amine oxidases: DAO, SSAO/VAP-1 and MAO-A and MAO-B. **c**, Proposed mechanism of lysyl oxidase inhibition by PXS-5505 through (i) initial binding to the LTQ complex in the enzymatic pocket to form a Schiff base, which then undergoes oxidation (ii) substitution of the fluorine with a nucleophilic amino acid (iii) formation of a covalently bound enzyme-inhibitor complex, resulting in irreversible loss of enzymatic activity. **d**, Representative plot of time-dependent inhibition of LOX specific activity showing increased potency with increased pre-incubation time, 0–240 min (*n* = 3 biologically independent samples). **e**, Representative plot of time-dependent inhibition of LOXL2 specific activity showing increased potency with increased pre-incubation time, 0–240 min (*n* = 3 biologically independent samples). **f**, IC_50_ values of PXS-5505 for each of the five lysyl oxidase family members. **g**, Lysyl oxidase family activity measured in the ear of rats following a single 30 mg kg^−1^ oral dosing of PXS-5505 *n* = 15 (PBS), 5 (4, 24 h), 4 (48, 120 h) biological independent samples. Data are presented as mean ± s.d. Two-tailed *P* value determined by unpaired, nonparametric *t*-test with a Mann–Whitney *U*-test correction (comparison to PBS control). **h**, Lysyl oxidase family activity measured in freshly excised aorta determined by fluorometric activity assay *n* = 19 (PBS), 5 (4–120 h) biological independent samples. Data presented as mean ± s.d. Two-tailed *P* value determined by unpaired, nonparametric *t*-test with a Mann–Whitney *U*-test correction (comparison to PBS control). Source data

To increase specificity for the lysyl oxidase family, the design of PXS-5505 (Supplementary Data 1–3) was based upon substrate analog fluoroallylamines. Variants of fluoroallylamines have previously been used in amine oxidase inhibitors, including those targeting LOXL2 and LOXL3 (ref. ^31^). Notably, small-molecule inhibitors based on the fluoroallylamine moiety are complete inhibitors of human LOXL2 enzymatic activity in vitro. Importantly, PXS-5505 not only maintains the potent functional inhibition of LOXL2 but linkage to a quinoline moiety improves potency for all other lysyl oxidase family members and is key to the superiority of PXS-5505 (Fig. 2a). PXS-5505 is the free base of the active principle, which is suitably isolated as a dihydrochloride salt. Crucially, PXS-5505 is not processed as a substrate for either SSAO or DAO (Extended Data Fig. 3a), and does not as an inhibitor of SSAO, DAO or the related monoamine oxidases A (MAO-A) and B (MAO-B) (Fig. 2b). Furthermore, PXS-5505 does not show any significant off-target activity against a standard panel of macromolecular targets at 30 µM (Eurofins SafetyScreen44; enzyme and radioligand-binding assays).

Similar to other fluoroallylamine-based amine oxidase inhibitors, the mode of inhibition is assumed to be a two-step mechanism (Fig. 2c), with PXS-5505 reacting with the unique LTQ cofactor found within the active site. The fluoroallylamine facilitates the formation of a covalent, irreversible bond. Evidence in support of this hypothesis includes substrate competition (Extended Data Fig. 3b), as well as the time-dependent nature of inhibition, with longer incubation times leading to increased potency (Fig. 2d,e). Furthermore, in jump dilution experiments, there is no significant recovery of activity following rapid dilution (LOXL1 (5 ± 5% recovery, *n* = 4), LOXL2 (7 ± 1.2% recovery, *n* = 3) and LOXL3 (11 ± 2% recovery, *n* = 3)).

In characterizing PXS-5505, the half-maximum inhibitory concentration (IC_50_) values for each of the lysyl oxidase family members were determined by measuring enzymatic activity using a fluorescence-based assay^32^. PXS-5505 showed low micromolar potency against all lysyl oxidase family members, with IC_50_ values ranging 0.2–5 µM (Fig. 2f and Extended Data Fig. 3c).

PXS-5505 displays a well-balanced absorption, distribution, metabolism and excretion (ADME) profile (Supplementary Table 2). Systemic exposure following oral administration shows high bioavailability (>75% for rodents) and short half-life (*~*1 h) (Extended Data Fig. 3d). In line with the high bioavailability of the small molecule, PXS-5505 penetrates core and peripheral matrix-rich tissues (the ear pinnae) well after oral administration (Extended Data Fig. 3e). PXS-5505 exhibits low plasma protein binding, high plasma stability, does not inhibit cytochrome P450 enzymes and exhibits high microsomal and hepatic stability, in agreement with low hepatic clearance relative to renal excretion (Supplementary Table 2). Overall PXS-5505 shows a very low propensity for any drug–drug interactions.

To confirm that PXS-5505 inhibits lysyl oxidase family enzymatic activity in tissues, target engagement experiments were performed following a single oral dose (30 mg kg^−1^) in rats. PXS-5505 showed the expected fast increase in plasma concentration followed by a rapid decay, with a calculated half-life of 1 h (Extended Data Fig. 3f). Lysyl oxidase family activity was measured in two tissues; aorta (core tissue) and ear (peripheral tissue). Activity in both tissues was significantly reduced 4 h after dosing (Fig. 2g,h) confirming that rapid penetration into cartilaginous tissues (ear) is occurring. The activity slowly recovered in the ear to approximately 50% over 120 h, whereas a faster recovery, to approximately 50% of initial activity was detectable in the aorta at 24 h after dosing (Fig. 2g,h). These data suggest that different tissue compartments have different de novo synthesis rates of lysyl oxidase family enzymes. Given the fast turnover in the aorta it is calculated that a single daily oral dose of 30 mg kg^−1^ PXS-5505 would block >70% of all lysyl oxidase family activity over a 24-h period.

We further confirmed PXS-5505 target engagement and inhibition of lysyl oxidase family activity in conditioned medium from cancer cells (CCs) and CAFs derived from the KPC mouse model (Extended Data Fig. 3g). In addition, we confirmed that at concentrations far exceeding lysyl oxidase family inhibition (up to 300 µM), PXS-5505 does not significantly alter transcription of lysyl oxidase family members in CCs or CAFs (Extended Data Fig. 3h) nor did it show any effect on proliferation in either two-dimensional (2D) (Extended Data Fig. 3i) or 3D (Extended Data Fig. 3j) settings.

PXS-5505 has successfully passed preclinical development, including genotoxicity and toxicity studies and is subject to two granted investigational new drug filings. These data have since enabled phase 1/2 clinical trials of PXS-5055 in myelofibrosis (NCT04676529).

### PXS-5505 blocks CAF-mediated crosslinking of collagen

**Fig. 3:**
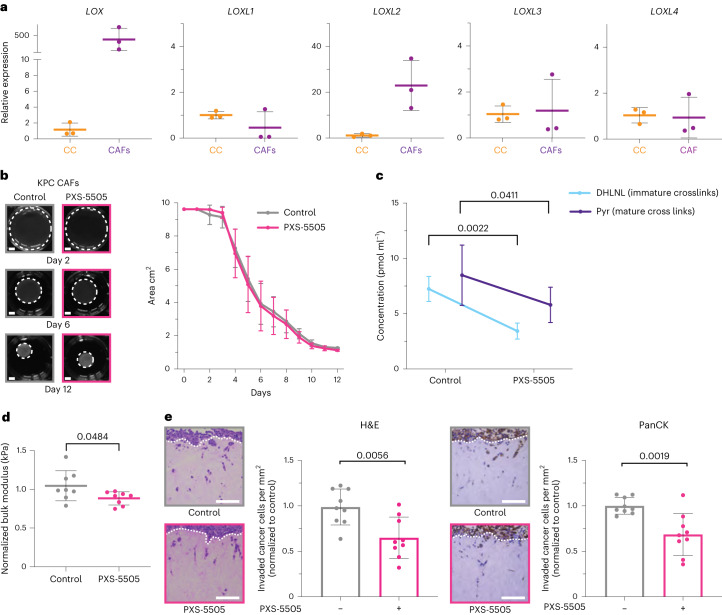
PXS-5505 inhibition of CAF remodeling in vitro. **a**, Quantitative PCR with reverse transcription (qRT–PCR) showing in vitro comparison of KPC CAF relative expression of lysyl oxidase family members compared to KPC CCs from the same model (*n* = 3 biologically independent samples). Comparisons were determined by 2^–∆∆*C*t^ approach. **b**, Representative images of 3D organotypic matrix contraction assay at days 2, 6 and 12 in the absence (gray) or presence (pink) of PXS-5505 (30 µM). Scale bars, 5 mm. Comparison of measured area of matrices between control and PXS-5505 treatment groups over time (*n* = 3 technical samples examined over three biologically independent samples). Data are mean ± s.d. **c**, Concentration of immature divalent DHLNL and mature trivalent Pyr collagen crosslinks determined by LC–MS from control and PXS-5505-treated organotypic matrices at day 12 (*n* = 3 technical samples examined over two biologically independent samples). Data are mean ± s.d. Two-tailed *P* value determined by unpaired, nonparametric *t*-test with a Mann–Whitney *U*-test correction (comparison between two groups). **d**, Comparison of matrix stiffness by unconfined compression testing of PXS-5505 and control treated matrices at day 12 (*n* = 8 biologically independent samples). Data are presented as mean ± s.d. Two-tailed *P* value determined by unpaired, nonparametric *t*-test with a Mann–Whitney *U*-test correction (comparison between two groups). **e**, Representative images taken from one biologically independently contracted matrix representative of the three biological replicates) of invasion of KPC CCs into a contracted organotypic matrix as determined by H&E and PanCK staining (±PXS-5505 at 30 µM during contraction phase only). Comparison of number of CCs invaded per field of view (FOV) as determined by H&E and PanCK staining (nine images taken from three technical samples from each of three biologically independent contracted matrices), normalized to control. Data are mean ± s.d. Scale bars, 100 μm. Two-tailed *P* value determined by unpaired, nonparametric *t*-test with a Mann–Whitney *U*-test correction (comparison between two groups). Source data

**Extended Data Fig. 4:**
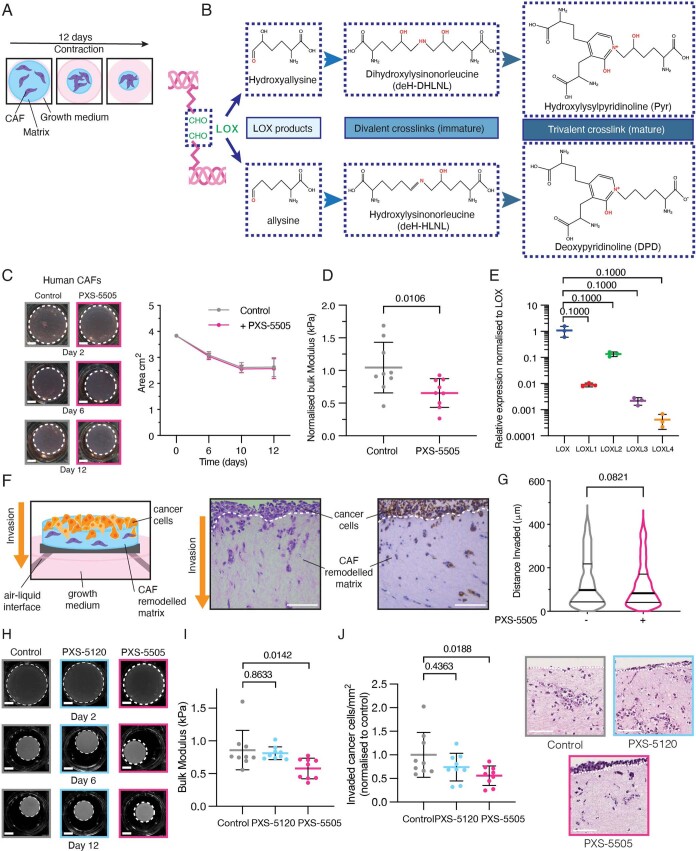
PXS-5505 inhibits collagen cross-linking by PDAC CAFs in vitro resulting in decreased cancer cell invasion. **a**. Schematic of organotypic matrix remodeling assay whereby CAFs are embedded into a 3D collagen matrix and growth media supplemented with or without PXS-5505 to inhibit lysyl oxidase family activity **b**. Chemical structures of lysyl oxidase family catalyzed hydroxyallysine or allysine residues and the subsequent divalent and trivalent bonds that are formed that can be quantified by mass spectrometry. **c**. Representative images of 3D organotypic matrix contraction by human pancreatic cancer CAFs at days 2, 6 and 12 in the absence (gray) and presence (pink) of PXS-5505 (30 µM). Scale bars = 5 mm. Comparison of area of matrices between control and PXS-5505 treatment groups (n = 3 biological repeats with 3 technical replicates per biological repeat) over time showing no effect on CAF ability to contract matrices. *p*-value determined by unpaired, nonparametric t-test with a Mann–Whitney U correction (comparison between two groups). Data presented as mean values +/− SD**. d**. Treatment with PXS-5505 during remodeling leads to a decrease in stiffness (bulk modulus) of remodeled organotypic matrices. *p*-value determined by unpaired, nonparametric t-test with a Mann–Whitney U correction (comparison between two groups). Data presented as mean values +/− SD**. e**. qRT-PCR confirming expression of lysyl oxidase family members from human PDAC CAFs (n = 3 biological repeats with 3 technical replicates per biological repeat). *p*-value determined by unpaired, nonparametric t-test with a Mann–Whitney U correction (comparison between two groups). Data presented as mean values +/− SD. Relative fold expression of LOXL family members compared to LOX were determined by 2^–∆∆Ct^ approach. **f**. Schematic of cancer cell invasion in a 3D organotypic matrix assay whereby cancer cells are seeded to the surface of a remodeled collagen plug and placed at an air–liquid interface and allowed to invade into the remodeled matrix. Example ROIs from H&E and panCK stained sections showing cancer cell invasion in the remodeled organotypic plug (3 biological repeats with 3 technical replicates per biological repeat). Scale bars = 100 μm. **g**. Comparison of invasion depth of cancer cells into 3D organotypic matrices as determined by PanCK staining n = -PXS-5505: 695 cells; +PXS-5505: 568 cells (taken from 3 technical replicates from 3 separate biological replicates), Data show distance invaded from the top of contracted matrix. *p*-value determined by unpaired, nonparametric t-test with a Mann–Whitney U correction (comparison between two groups). **h**. Representative images of 3D organotypic matrix contraction assay at days 2, 6 and 12 of control (gray) and presence of PXS-5120 (blue) and PXS-5505 (pink). Scale bars = 5 mm. Comparison of measured area of matrices between control, PXS-5120 and PXS-5505 treatment groups (n = 3) over time. **i**. Comparison of matrix stiffness by unconfined compression testing of PXS-5120, PXS-5505 and control treated matrices at day 12 (n = 3 biological repeats with 3 technical replicates per biological repeat). Data presented as mean values +/− SD. *p*-value determined by unpaired, nonparametric t-test with a Mann–Whitney U correction (comparison between two groups) **j**. Representative images of invasion of KPC cancer cells into a contracted organotypic matrix as determined by H&E staining. Comparison of number of cancer cells invaded per FOV as determined by H&E (9 images from 3 biological repeats with 3 technical replicates per biological repeat), shown as cells/mm^2^ and normalized to control (n = 9). Data presented as mean values +/− SD. *p*-value determined by unpaired, nonparametric t-test with a Mann–Whitney U correction (comparison between two groups). Source data

To determine the effects of blocking lysyl oxidase family-mediated collagen crosslinking in vitro and the subsequent effects on pancreatic CCs, we used our 3D organotypic co-culture model, where KPC-derived CAFs^28^ are embedded into and allowed to remodel 3D collagen I matrices (Extended Data Fig. 4a). Expression of lysyl oxidase family members was confirmed in CCs and CAFs derived from the KPC model, as well as the ability of PXS-5505 to inhibit them (Fig. 3a and Extended Data Fig. 3g).

KPC CAFs embedded in 3D organotypic matrices remodel and crosslink them. Treatment with PXS-5505 during the remodeling phase showed no macroscopic effect on matrix contraction (Fig. 3b). Liquid chromatography–mass spectrometry (LC–MS) quantification of collagen crosslinks, the product of lysyl oxidase activity, showed decreases in immature dihydroxylysinonorleucine (DHLNL) and mature hydroxylysylpyridinoline (Pyr) (normalized to total collagen content; hydroxyproline abundance) (Fig. 3c and Extended Data Fig. 4b) upon PXS-5505 administration confirming PXS-5505-target engagement and inhibition.

Biomechanical validation of the effect of collagen crosslinking inhibition by PXS-5505 on organotypic matrices was carried out by using unconfined compression analysis. PXS-5505 decreased CAF-mediated collagen stiffening (Fig. 3d), supporting the proteomic quantification of decreased crosslink number. We further validated these findings in a second model using human primary pancreatic cancer CAFs^33^. We verified lysyl oxidase family expression in these CAFs before confirming PXS-5505 also inhibited collagen crosslinking and stiffening in this model (Extended Data Fig. 4c–e). Together these data demonstrate PXS-5505 alters both murine and human PDAC CAF ability to remodel, crosslink and stiffen the 3D collagen matrix.

### PXS-5505 decreases pancreatic cancer cell invasion

To dissect the physiological effects of differentially crosslinked collagen matrices on cancer cell behavior, KPC CCs were seeded onto CAF-remodeled organotypic matrices (Extended Data Fig. 4f), placed at an air–liquid interface and allowed to invade^28,34^. Inhibition of lysyl oxidases by PXS-5505 led to a decrease in invasion into the organotypic matrices by CCs (Extended Data Fig. 4f). Where CC invasion did occur, there was no significant difference in depth of invasion (Fig. 3e and Extended Data Fig. 4g).

Effects of PXS-5505 inhibition were also compared to a dual LOX/LOXL2 small-molecule inhibitor PXS-5120 (PXS-S2A^35^). These data demonstrate that PXS-5120 does not significantly block stiffening of 3D collagen matrices and consequently does not lead to decreased CC invasion compared to the pan-lysyl oxidase inhibitor, PXS-5505. (Extended Data Fig. 4h–j).

### PXS-5505 plus gemcitabine significantly improves survival in vivo

**Fig. 4:**
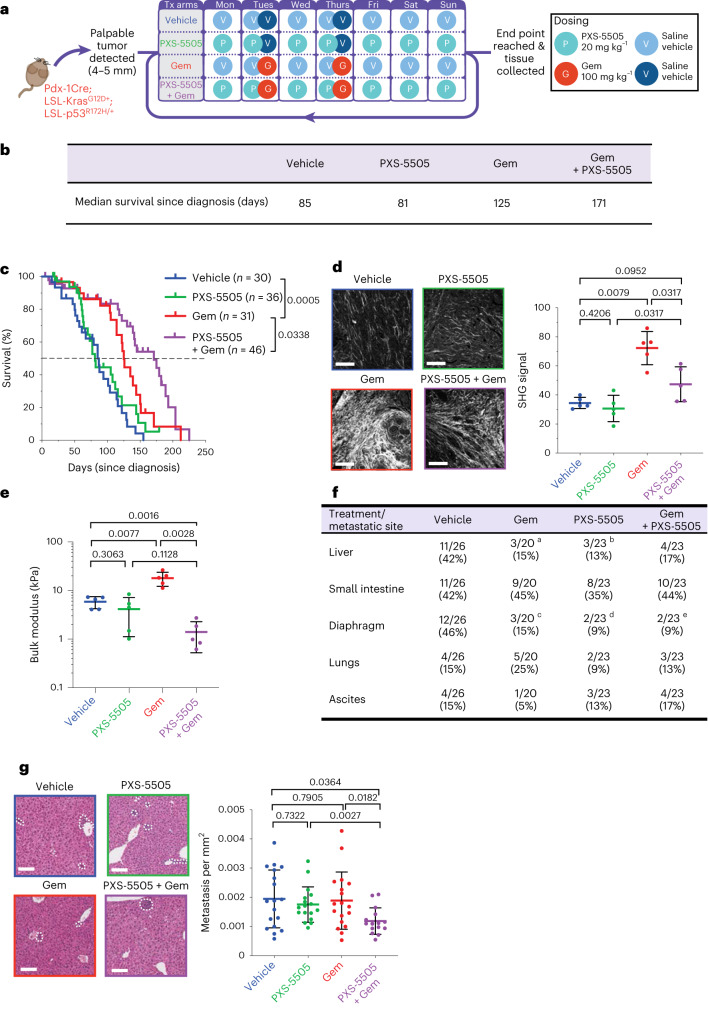
PXS-5505 administered in a KPC in vivo survival study. **a**, Schematic of the KPC in vivo survival study. Presence of a primary tumor in KPC mice was confirmed by two independent researchers (positive diagnosis) before commencement of daily treatment (Tx) on one of four treatment arms as follows: vehicle, 0.9% saline; Gem, twice-weekly gemcitabine (100 mg kg^−1^ i.p.); PXS-5505, daily PXS-5505 at 20 mg kg^−1^ i.p.; and PXS-5505 + Gem, daily PXS-5505 at 20 mg kg^−1^ i.p. + twice-weekly gemcitabine (100 mg kg^−1^ i.p.). **b**, Table of median survival (since diagnosis/commencement of treatment) across the four treatment groups (*n* = 30 biologically independent animals (vehicle), *n* = 31 biologically independent animals (Gem), *n* = 36 biologically independent animals (PXS-5505), *n* = 46 biologically independent animals (PXS-5505 + Gem)). **c**, Kaplan–Meier curves for overall survival across treatment arms (*n* = 30 biologically independent animals (vehicle), *n* = 31 biologically independent animals (Gem), *n* = 36 biologically independent animals (PXS-5505), *n* = 46 biologically independent animals (PXS-5505 + Gem)). *P* values determined by log-rank (Mantel–Cox) test. **d**, Representative maximum intensity projections of SHG multiphoton imaging for tumors from each treatment group (*n* = 1 FOV taken from one biologically independent animal per group) at end point (scale bars, 100 μm) and quantification of SHG peak signal intensity (*n* = 5 biologically independent animals per group and five FOV per animal). Data are mean ± s.d. Two-tailed *P* values determined by unpaired, nonparametric *t*-test with a Mann–Whitney *U*-test correction (comparison between two groups) **e**, Quantification of bulk modulus (stiffness) by unconfined compression testing of end-point PDAC tumors from each treatment group (*n* = 5 biologically independent animals per group; individual tumors shown), data are mean ± s.d. Two-tailed *P* value determined by unpaired, nonparametric *t*-test with a Mann–Whitney *U*-test correction (comparison between two groups). **f**, Presence of overt metastatic lesions observed during necropsy (number of mice). Two-tailed *P* values determined by chi-squared test. ^a^Liver (vehicle versus Gem, *P* = 0.046, chi-squared); ^b^Liver (vehicle versus PXS-5505, *P* = 0.023, chi-squared); ^c^Diaphragm (vehicle versus Gem, *P* = 0.026, chi-squared); ^d^Diaphragm (vehicle versus PXS-5505, *P* = 0.004, chi-squared); and ^e^Diaphragm (vehicle versus Gem + PXS-5505, *P* = 0.004, chi-squared). **g**, Representative images of H&E-stained livers from each treatment group (*n* = 1 FOV taken from one biologically independent animal per group) (scale bars, 100 μm). Quantification of metastases (*n* = 18 biologically independent animals (vehicle), *n* = 18 biologically independent animals (Gem), *n* = 17 biologically independent animals (PXS-5505), *n* = 15 biologically independent animals (PXS-5505 + Gem)). Data are mean ± s.d. Two-tailed *P* values were determined by unpaired, nonparametric *t*-test with a Mann–Whitney *U*-test correction (comparison between two groups). Source data

We next evaluated the effects of PXS-5505 in vivo in combination with the clinically approved chemotherapy agent, gemcitabine (Fig. 4a). We hypothesized that a pan-lysyl oxidase inhibitor would improve outcome when used in combination with chemotherapy, through blunting the development of tumor desmoplasia.

**Extended Data Fig. 5:**
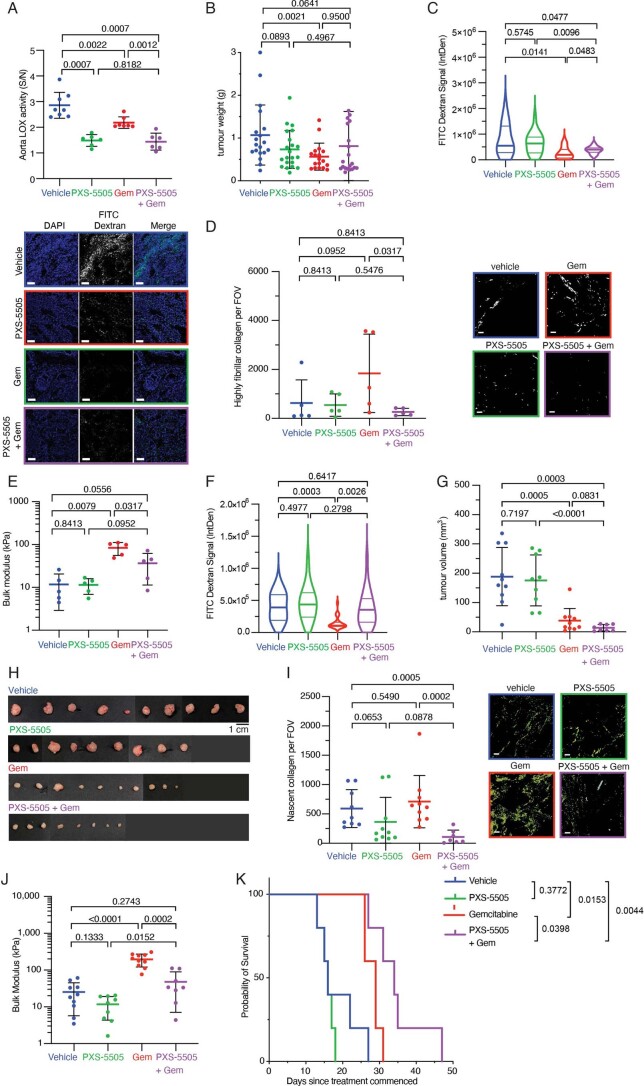
PXS-5505 in combination with gemcitabine *in vivo*. **a**. Lysyl oxidase family activity measured in the aorta of KPC tumor bearing mice at end point, measured by fluorometric activity assay confirming lysyl oxidase family inhibition. Signal is defined as the enzyme activity in the aorta, while noise is measured in the presence of a high concentration (300 µM) BAPN to abolish any lysyl oxidase family activity. Signal over noise equal to 1 indicates complete inhibition. *p*-value determined by unpaired, nonparametric t-test with a Mann–Whitney U correction (comparison to vehicle control) (vehicle n = 8 biologically independent animals, Gem n = 7 biologically independent animals, PXS-5505 n = 6 biologically independent animals, PXS-5505 + Gem n = 6 biologically independent animals). Data presented as mean values +/− SD **b**. Tumor weight (grams) at end point from each treatment group (vehicle n = 19 biologically independent animals, Gem n = 18 biologically independent animals, PXS-5505 n = 21 biologically independent animals, PXS-5505 + Gem n = 21 biologically independent animals). *p*-value determined by unpaired, nonparametric t-test with a Mann–Whitney U correction (comparison between two groups). Data presented as mean values +/− SD. **c**. FITC-dextran (molecular weight: 10 kDa) signal quantification from KPC tumor end point from each treatment group (vehicle n = 3 biologically independent animals, PXS-5505 n = 5 biologically independent animals, Gem n = 5 biologically independent animals, PXS-5505 + Gem n = 5 biologically independent animals). 4 ROIs taken per mouse. *P*-values determined by Welchs t-test. Data presented as mean values +/− SD. Scale bars = 100 μm **d**. Quantification of red birefringent (mature) collagen fibers from human PDXs at end point after 3 cycles of treatment, treated as indicated. 5 fields of view (500 μm × 500 μm) per mouse. *p*-values determined by unpaired, nonparametric t-test with a Mann–Whitney U correction (vehicle n = 5 biologically independent animals, Gem n = 5 biologically independent animals, PXS-5505 n = 5 biologically independent animals, PXS-5505 + Gem n = 5 biologically independent animals). Data presented as mean values +/− SD. Scale bars = 100 μm **e**. Quantification of bulk modulus (stiffness) by unconfined compression testing of end point human PDX tumors from each treatment group (vehicle n = 5 biologically independent animals, Gem n = 5 biologically independent animals, PXS-5505 n = 5 biologically independent animals, PXS-5505 + Gem n = 5 biologically independent animals) [individual tumors shown]). Data presented as mean values +/− SD. *p*-value determined by unpaired, nonparametric t-test with a Mann–Whitney U correction (comparison between two groups) **f**. FITC-dextran (molecular weight 10 kDa) signal quantification from human PDXs from each treatment group (vehicle n = 4 biologically independent animals, PXS-5505 n = 5 biologically independent animals, Gem n = 5 biologically independent animals, PXS-5505 + Gem n = 5 biologically independent animals). 4 ROIs taken per mouse. P values determined by Welchs t-test. **g**. A second human PDX model at end point after 3 cycles of treatment. Tumor volumes (mm^3^) at end point from each treatment group (vehicle n = 9 biologically independent animals, Gem n = 10 biologically independent animals, PXS-5505 n = 10 biologically independent animals, PXS-5505 + Gem n = 7 biologically independent animals). *p*-value determined by unpaired, nonparametric t-test with a Mann–Whitney U correction (comparison between two groups). Data presented as mean values +/− SD. **h**. Image of human PDX tumors after 3 cycles of treatment **i**. Quantification of green birefringent (nascent) collagen fibers from human PDXs at end point, treated as indicated. 3 fields of view (750 μm × 750 μm) per mouse. *p*-values determined by unpaired, nonparametric t-test with a Mann–Whitney U correction (vehicle n = 10 biologically independent animals, Gem n = 10 biologically independent animals, PXS-5505 n = 9 biologically independent animals, PXS-5505 + Gem n = 7 biologically independent animals). Data presented as mean values +/− SD. Scale bars = 100 μm **j**. Quantification of bulk modulus (stiffness) by unconfined compression testing of end point human PDX tumors from each treatment group (vehicle n = 10 biologically independent animals, Gem n = 10 biologically independent animals, PXS-5505 n = 9 biologically independent animals, PXS-5505 + Gem n = 8 biologically independent animals) [individual tumors shown]). Data presented as mean values +/− SD. *p*-value determined by unpaired, nonparametric t-test with a Mann–Whitney U correction (comparison between two groups) **k**. Kaplan–Meier curves for overall survival across treatment arms (n = 5 (vehicle), n = 5 (Gem), n = 5 (PXS-5505), n = 5 (PXS-5505 + Gem). Treatment commenced once tumor reached 50 mm^3^ *p*-values determined by Log-rank (Mantel–Cox) test. Source data

KPC mice were treated from the time at which palpable tumors were detected (typically 10 to 12 weeks of age) and continued until the study end point. Mice were treated daily with PXS-5505 (20 mg kg^−1^ i.p.) or vehicle (0.9% saline) and twice weekly with gemcitabine (100 mg kg^−1^ i.p.) or vehicle (0.9% saline). To confirm inhibition of lysyl oxidase family activity, aortas of mice were collected and lysyl oxidase activity measured to confirm PXS-5505 target inhibition (Extended Data Fig. 5a).

Treatment of KPC mice with gemcitabine led to a slowing of primary tumor progression, increasing survival from a median survival of 85 days in untreated mice to 125 days in gemcitabine-treated mice (*P* = 0.0005) (Fig. 4b,c, blue versus red). PXS-5505 as a monotherapy led to no changes in median survival compared to vehicle (85 versus 81 days) (Fig. 4c, blue versus green). Notably, the combination therapy of PXS-5505 daily plus gemcitabine twice weekly led to a statistically significant increase in median survival compared to gemcitabine alone (171 versus 125 days, *P* = 0.0338) (Fig. 4b,c, red versus purple). At the end point, tumor weights showed small decreases in each of the treated groups compared to vehicle, but no significant differences between treatments (Extended Data Fig. 5b). These data strongly support that addition of PXS-5505 to gemcitabine potentiates the efficacy of chemotherapy and this study provides evidence that a pan-lysyl oxidase small-molecule-targeting approach can significantly improve gemcitabine efficacy.

SHG multiphoton imaging analysis of end-point tumors for fibrillar collagen type showed that gemcitabine treatment, while extending survival, led to significantly increased stromal content within the tumors (Fig. 4d). Critically, in mice treated with the combination of gemcitabine + daily PXS-5505, there was a significant reduction in total fibrillar collagen content within tumors (Fig. 4d). To investigate how these altered levels of tumor desmoplasia might be affecting tumor perfusion, fluorescein isothiocyanate (FITC) dextran (molecular mass of 10 kDa corresponding to the size of gemcitabine) was intravenously perfused before culling and used to track delivery. Gemcitabine monotherapy significantly reduced FITC signal compared to control (Extended Data Fig. 5c), indicative of decreased perfusion into the tumor and in line with the consensus that high levels of tumor desmoplasia limit entry of agents. Dual treatment with PXS-5505 plus gemcitabine increased the FITC signal compared to gemcitabine alone, confirming higher levels of perfusion and suggesting that delivery into the tumor is improved in the combination treatment group underpinning the observed extended survival (*P* = 0.0483) (Extended Data Fig. 5c). This is particularly noteworthy given the extended survival time of the combination therapy group.

To further confirm the effects of PXS-5505 on the biomechanical properties of tumors, unconfined compression analysis was undertaken (Fig. 4e). These data show that in addition to increasing collagen content (measured by SHG and picrosirius red), gemcitabine also leads to increases in tumor bulk modulus (stiffness) by approximately threefold (vehicle 5.9 kPa, gemcitabine 20.0 kPa; *P* = 0.0077). PXS-5505 alone showed a moderate reduction in stiffness compared to vehicle. PXS-5505 in combination with gemcitabine led to a significant decrease in tumor bulk modulus compared to gemcitabine alone (gemcitabine 20.0 kPa, gemcitabine + PXS-5505 1.4 kPa; *P* = 0.0028) (Fig. 4e). These data are in line with decreases in stromal fibrillar collagen content (Fig. 4d) confirming that inhibition of lysyl oxidase family activity in combination with chemotherapy leads to a reduction in stromal collagen content, decreases in tumor stiffness and improved perfusion, which likely contribute to the potentiation of gemcitabine efficacy in the KPC mouse model of PDAC.

Next, we sought to confirm the effects of PXS-5505 on human pancreatic tumor stroma using two complementary human PDX models. The TKCC10 model from the APGI cohort is an untreated, grade 3 tumor that expresses high levels of lysyl oxidase family members^21,36^. Following implantation, this model was treated with three rounds of therapy (Fig. 4a) (matched time point study). Tumors were excised and picrosirius red birefringence analysis carried out confirming that gemcitabine increases fibrillar collagen I content within the tumor (Extended Data Fig. 5d). Similar to the KPC model, the combination of gemcitabine + PXS-5505 led to a significant decrease in tumor fibrillar collagen content. Furthermore, unconfined compression analysis measurements confirmed that gemcitabine led to a significant increase in tumor bulk stiffness that was blunted by combination treatment with PXS-5505 (Extended Data Fig. 5e). Like the KPC model, FITC-dextran perfusion showed that gemcitabine significantly reduced FITC signal compared to control (Extended Data Fig. 5f), indicative of decreased perfusion into the tumor; however, dual treatment with the combination of PXS-5505 + gemcitabine significantly increased the FITC signal compared to gemcitabine alone, confirming higher levels of perfusion in the tumor compartment and suggesting that delivery of gemcitabine into the tumor is improved in the combination treatment group (Extended Data Fig. 5f).

A second human PDX model from the Australian Pancreatic Cancer Matrix Atlas (APMA) was also evaluated. Implanted PDX tumors (carrying confirmed mutations in *KRAS*, *TP53*, *CDKN2A*, *ARID1A*, *MLL2*, *MLL3* and *KDM6A*) underwent three rounds of treatment (Fig. 4a) (matched time point study). Following tumor excision (Extended Data Fig. 5g,h), picrosirius red birefringence analysis confirmed an increase in the deposition of collagen I into the tumor microenvironment as a result of gemcitabine administration in this model (Extended Data Fig. 5i), matching data from the KPC mouse and TKCC10 human PDX models. Gemcitabine also led to an increase in tumor stiffness (Extended Data Fig. 5j). Both increases were blunted in the combination PXS-5505 + gemcitabine treatment group. Together these data confirm that PXS-5505 is attenuating the tumor desmoplastic response and, in particular, therapy-induced fibrosis in both mouse and human models of PDAC.

A separate survival study using the TKCC10 human model was also performed with treatment as per the treatment schedule outlined in Fig. 4a. In this study both the vehicle-treated and PXS-5505-treated mice had a median survival of 16 d (measured as the time under treatment after reaching a minimum tumor size threshold of 50 mm^3^) and mimicking the survival patterns seen in the original KPC mouse model. Gemcitabine alone extended median survival to 29 d in these mice and PXS-5505 combination with gemcitabine further extended this to 34 days (Extended Data Fig. 5k).

**Extended Data Fig. 6:**
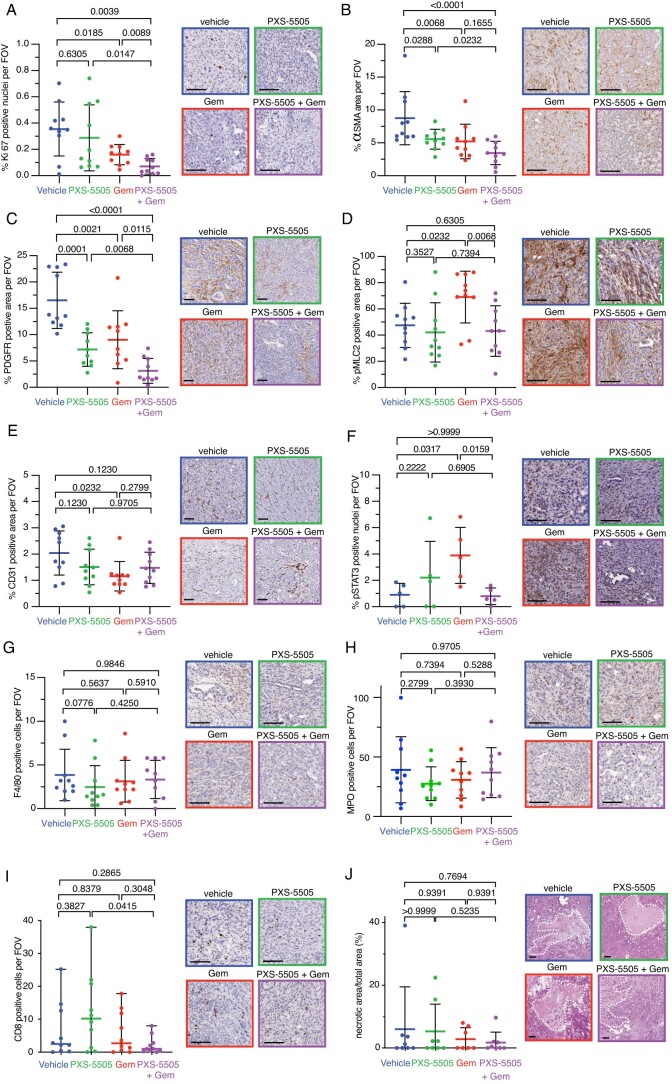
PXS-5505 in combination with gemcitabine in a KPC model. **a**. Quantification of proliferating cells (Ki67 positive nuclear staining) in KPC tumors at end point, treated as indicated. 5 fields of view (500 μm × 500 μm) per mouse and 10 mice per cohort were scored. Data presented as mean values +/− SD. *p*-values determined by unpaired, nonparametric t-test with a Mann–Whitney U correction. Scale bars = 100 μm **b**. Quantification of percent coverage of αSMA positive staining in KPC tumors at end point, treated as indicated. 3 fields of view (1000 μm × 1000 μm) per mouse and 10 mice per cohort were scored. Data presented as mean values +/− SD. *p*-values determined by unpaired, nonparametric t-test with a Mann–Whitney U correction. Scale bars = 100 μm **c**. Quantification of percent coverage of PDGFR-ß positive staining in KPC tumors at end point, treated as indicated. 3 fields of view (1000 μm × 1000 μm) per mouse and 10 mice per cohort were scored. Data presented as mean values +/− SD. *p*-values determined by unpaired, nonparametric t-test with a Mann–Whitney U correction. Scale bars = 100 μm **d**. Quantification of pMLC2 positive staining in stromal regions of KPC tumors at end point, treated as indicated. 3 fields of view (500 μm × 500 μm) per mouse and 10 mice per cohort were scored. Data presented as mean values +/− SD. *p*-values determined by unpaired, nonparametric t-test with a Mann–Whitney U correction. Scale bars = 100 μm **e**. Quantification of tumor vasculature (CD31 positive staining) in KPC tumors at end point, treated as indicated. 3 fields of view (1000 μm × 1000 μm) per mouse and 10 mice per cohort were scored. Data presented as mean values +/− SD. *p*-values determined by unpaired, nonparametric t-test with a Mann–Whitney U correction. Scale bars = 100 μm **f**. Quantification of STAT3 activation (pSTAT3 positive nuclear staining) in KPC tumors at end point, treated as indicated. 3 fields of view (1000 μm × 1000 μm) per mouse and 5 mice per cohort were scored. Data presented as mean values +/− SD. *p*-values determined by unpaired, nonparametric t-test with a Mann–Whitney U correction. Scale bars = 100 μm. **g** Quantification of macrophage infiltration (F4/80 positive staining) in KPC tumors at end point, treated as indicated. 5 fields of view (500 μm × 500 μm) per mouse and 10 mice per cohort were scored. Data presented as mean values +/− SD. *p*-values determined by unpaired, nonparametric t-test with a Mann–Whitney U correction. Scale bars = 100 μm **h**. Quantification of number of myeloperoxidase (MPO^+^) cell infiltration in KPC tumors at end point, treated as indicated. 5 fields of view (500 μm × 500 μm) per mouse and 10 mice per cohort were scored. Data presented as mean values +/− SD. *p*-values determined by unpaired, nonparametric t-test with a Mann–Whitney U correction. Scale bars = 100 μm **i**. Quantification of T-cell infiltration (CD8 positive staining) in KPC tumors at end point, treated as indicated. 5 fields of view (500 μm × 500 μm) per mouse and 10 mice per cohort were scored. *p*-values determined by unpaired, nonparametric t-test with a Mann–Whitney U correction. Data presented as median values with range. Scale bars = 100 μm **j**. Quantification of necrosis determined by H&E in KPC tumors at end point, treated as indicated (vehicle n = 8 biologically independent animals, Gem n = 7 biologically independent animals, PXS-5505 n = 8 biologically independent animals, PXS-5505 + Gem n = 8 biologically independent animals). Data presented as mean values +/− SD. *p*-values determined by unpaired, nonparametric t-test with a Mann–Whitney U correction. Scale bars = 100 μm. Source data

To further characterize the effects of PXS-5505 on the tumor microenvironment, we characterized several key markers in the KPC survival study tumors. Ki67 staining (cancer cell proliferation) (Extended Data Fig. 6a) confirmed that at the end point, gemcitabine is significantly decreasing the number of Ki67 positive tumor cells (*P* = 0.0185) and that this is further decreased in combination with PXS-5505 (*P* = 0.0089), underpinning the significantly decreased tumor growth observed in the combination therapy arm, likely through sustained perfusion of agents into the tumor.

Staining for α-smooth muscle actin (α-SMA) (myCAF marker) and platelet derived growth factor receptor-β (PDGFR-β) (panCAF marker) (Extended Data Fig. 6b,c) showed small decreases in positivity (*P* = 0.0068) and (*P* = 0.0021), respectively in the gemcitabine-treated mice and subsequent further decreases (*P* = 0.1655 α-SMA and *P* = 0.0115 PDGFR-β) in the PXS-5505 plus gemcitabine combination-treated mice.

Staining for phospho-myosin light chain 2 (pMLC2) in stromal regions (a marker of stiffness induced CAF activation and contractility) showed increased pMLC2 in gemcitabine-treated (stiffer) tumors, which was significantly decreased in combination-treated (softer) tumors (Extended Data Fig. 6d), matching the observed changes in tumor stiffnesses (Fig. 4e). Gemcitabine appeared to moderately reduce CD31 positivity within tumors, though this was not further affected in combination with PXS-5505 (Extended Data Fig. 6e).

To investigate the intracellular mechanisms, KPC tumors were also stained for phosphoSTAT3 (Extended Data Fig. 6f), an important regulator of cancer cell survival^37^ that is upregulated in PDAC. STAT3 signaling is known to be altered by tissue stiffness, especially within the tumor context. Furthermore, increased STAT3 signaling has been linked to chemoresistance in pancreatic cancer and targeting STAT3 signaling synergizes with gemcitabine chemotherapy in mouse models of PDAC^38^. Gemcitabine-treated mice exhibited significantly increased levels of pSTAT3-positive staining (*P* = 0.0317) compared to vehicle (determined as % of pSTAT3-positive nuclear staining); however, combination treatment of gemcitabine + PXS-5505 significantly decreased pSTAT3 staining (*P* = 0.0159). These data suggest that the PXS-5505-mediated decreases in tumor desmoplasia and stiffness lead to a decrease in activation of pSTAT3 signaling in cancer cells, a known critical mediator of anti-apoptotic pathways and tumor progression^39–42^. Finally, immunohistochemical analysis revealed that there were no statistically significant differences in infiltration of F4/80^+^ (myeloid), MPO^+^ (neutrophil/eosinophil/monocytic) or CD8^+^ infiltration between any of the treatment groups at the end point (Extended Data Fig. 6g–i). We also observed no significant changes in macroscopic tumor necrosis between treatment groups in these endpoint tumours (Extended Data Fig. 6j).

Together, these in vivo data indicate that addition of PXS-5505 to gemcitabine, blunts the development of chemotherapy-induced desmoplasia, decreases tumor stiffness, improves perfusion of agents into tumors, restricts the associated stromal activation of CAFs and decreases pSTAT3 activation in pancreatic cancer cells, thereby augmenting the efficacy of gemcitabine therapy and extending survival in these mice.

### PXS-5505 plus gemcitabine reduces metastasis in KPC mice

KPC mice expressing mutant p53^R172H^ are known to exhibit a high frequency of metastasis to a number of organs and in particular the liver^43,44^. We quantified presence of overt metastatic dissemination to organs across the different treatment groups (Fig. 4f).

PXS-5505 as a monotherapy, while not extending overall survival, decreased the presence of overt metastatic disease in peritoneal organs of these mice (Fig. 4f) compared to vehicle, highlighting a potential anti-metastatic effect. Furthermore, in addition to significantly extending median survival, the combination of PXS-5505 plus gemcitabine showed a similar presence of overt metastatic disease in organs compared to gemcitabine alone, despite the significantly increased timeframe for metastatic dissemination to occur.

We undertook further microscopic quantification of the metastatic burden in the liver, a primary site of PDAC metastasis. Quantification of metastases revealed that gemcitabine did not reduce the number of metastatic foci, nor did PXS-5505 alone, compared to vehicle (Fig. 4g); however, the combination treatment of PXS-5505 plus gemcitabine resulted in a significant decrease in metastatic burden within the liver (Fig. 4g). This is particularly pertinent given that these mice survived longer and thus experienced a significantly longer time window for metastasis to occur. In addition, we saw no evidence of chemotherapy-induced liver fibrosis in this model; however, we could not rule out that PXS-5505 may be having a small effect on chemotherapy-induced liver fibrosis, which may be contributing to the reduced metastatic burden.

Together, our in vitro and in vivo data demonstrate that PXS-5505 inhibition of lysyl oxidase family-mediated collagen crosslinking combined with chemotherapy reduces tumor desmoplasia, decreases local invasion through a collagen-rich environment and reduces metastasis in vivo.

### PXS-5505 reduces early metastatic colonization of the liver

Our in vivo data suggest that PXS-5505 may be influencing the survival and overt colonization of disseminating tumor cells at secondary metastatic sites. We sought to determine the effects of PXS-5505 in combination with gemcitabine on metastatic colonization of the liver at matched time points. To do so, we used the intrasplenic model of hepatic colonization^28,34^. This orthotopic model of liver metastasis bypasses early steps of metastasis (local invasion and intravasation) allowing for assessment of the effects of an intervention specifically on secondary tissue metastatic colonization.

**Fig. 5:**
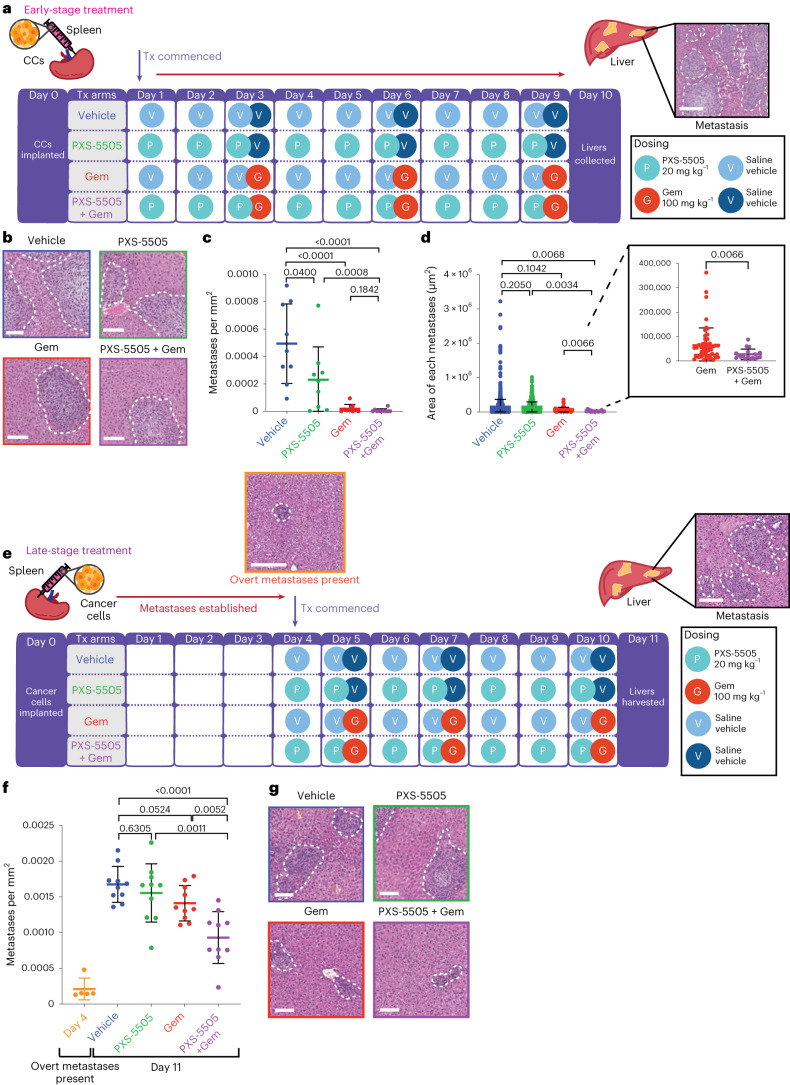
PXS-5505 administered in intrasplenic models of liver colonization. **a**, Schematic of the intrasplenic model of liver colonization with early administration of treatment. CCs were implanted into the spleen of BALB/c-Fox1nuAusb mice under anesthesia on day 0. Treatment with 0.9% saline (vehicle); twice-weekly Gem (100 mg kg^−1^ i.p.) (Gem); daily PXS-5505 at 20 mg kg^−1^ i.p. (PXS-5505) or daily PXS-5505 at 20 mg kg^−1^ i.p. + twice-weekly Gem at 100 mg kg^−1^ i.p. (PXS-5505 + Gem) was administered from day 1. At the end point (day 10) livers were collected and H&E-stained for scoring. Representative images of H&E-stained liver (taken from one biologically independent animal). Scale bar, 100 μm. **b**, Representative images of H&E-stained livers from each treatment group (*n* = 1 FOV taken from one biologically independent animal per group). Scale bars, 100 μm. **c**, Quantification of metastases per mm^2^ (*n* = 9 biologically independent animals per group). Data are mean ± s.d. Two-tailed *P* values were determined by unpaired, nonparametric *t*-test with a Mann–Whitney *U*-test correction (comparison between two groups). **d**, Quantification of the area of each metastasis in treatment groups (*n* = 9 biologically independent animals per group). Data are mean ± s.d. Two-tailed *P* values were determined by an unpaired, nonparametric *t*-test with a Mann–Whitney *U*-test correction (comparison between two groups). **e**, Schematic of intrasplenic model of liver colonization with late administration of treatment. CCs were implanted into the spleen of BALB/c-Fox1nuAusb mice under anesthesia on day 0. Treatment with 0.9% saline (vehicle); twice-weekly Gem (100 mg kg^−1^ i.p.) (Gem); daily PXS-5505 at 20 mg kg^−1^ i.p. (PXS-5505); or daily PXS-5505 at 20 mg kg^−1^ i.p. + three doses of Gem (100 mg kg^−1^ i.p. (PXS-5505 + Gem) was administered from day 4. Representative images of H&E-stained liver (taken from one biologically independent animal). Scale bar, 100 μm. **f**, Quantification of metastases at endpoint (*n* = 10 biologically independent animals per treatment group, *n* = 5 biologically independent animals at day 4 for confirmation of overt metastases). Data are mean ± s.d. *P* values were determined by an unpaired, nonparametric *t*-test with a Mann–Whitney *U*-test correction (comparison between two groups). **g**, Representative images of H&E-stained livers from each treatment group. Scale bars, 100 μm. Source data

KPC tumor cells were inoculated into the spleen (day 0) and treated from day 1 as per (Fig. 5a) until day 10. At the end point, livers were collected, fixed and step sections were scored by hematoxylin and eosin (H&E) for metastatic burden. Treatment with gemcitabine significantly decreased metastatic colonization compared to the vehicle, leading to both fewer (Fig. 5b,c) and smaller (Fig. 5b,d) metastatic foci. Notably, PXS-5505 alone also led to a small decrease in number (Fig. 5b,c), but not size of metastatic foci (Fig. 5b,d) suggesting that the activity of the lysyl oxidase family may be critical in the early stages of colonization. Most notable was the significant decrease in average size of metastatic foci in the combination (PXS-5505 + gemcitabine) treated mice, compared to gemcitabine alone (Fig. 5b,d, inset). These data, combined with effects seen on spontaneous metastatic dissemination in the KPC autochthonous model, further support our hypothesis that PXS-5505 can potentiate the efficacy of gemcitabine chemotherapy in vivo in the metastatic setting.

### PXS-5505 enhances response of established metastases to chemotherapy

Many patients with PDAC present in the clinic with already established metastatic disease, which limits treatment options. Systemic chemotherapy, such as gemcitabine, is usually the only option available to patients with stage IV metastatic pancreatic cancer and the refractive nature of metastatic PDAC to many therapies is a significant clinical challenge. We sought to determine whether PXS-5505 had the potential to increase the efficacy of gemcitabine against established metastatic disease. To do so, we repeated the intrasplenic liver colonization assay, this time commencing treatment after overt metastatic disease had been confirmed (Fig. 5e).

**Extended Data Fig. 7:**
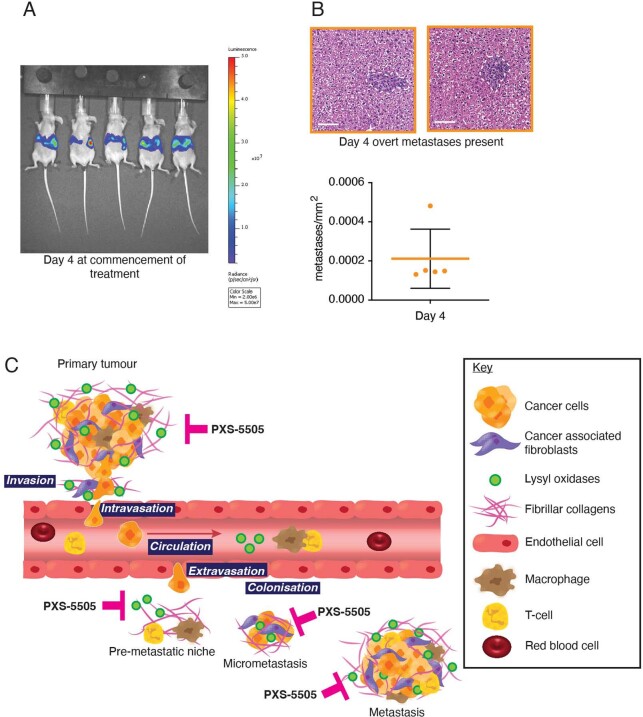
Liver colonization in the intrasplenic model and schematic of PXS-5505 inhibition of lysyl oxidases in PDAC. **a**. IVIS imaging of late-stage treatment study of liver colonization model over 11 days. **b**. Representative images of H&E-stained liver at day 4 confirming presence of micro-metastatic lesions as detected by IVIS imaging n = 5 independent animals shown in (A). Data presented as mean values +/− SD. Scale bar 100 μm. Data also shown in Fig. 5f (orange) for reference **b**. PXS-5505 inhibits lysyl oxidase family member activity at different stages of tumor progression and metastasis. Source data

Treatment began 4 d after inoculation when overt metastatic disease was present (confirmed by IVIS bioluminescent imaging, (Extended Data Fig. 7a) and H&E analysis (Fig. 5e inset and Extended Data Fig. 7b). H&E analysis of liver sections (confirmed using CK19 staining), revealed at the end point that gemcitabine or PXS-5505 alone did not significantly alter metastatic burden (Fig. 5f,g); however, PXS-5505 + gemcitabine combination therapy led to a small but significant decrease in metastatic burden compared to all other groups (Fig. 5f,g), indicating that PXS-5505 may be beneficial in combination with chemotherapy in the late-stage metastatic setting in patients where surgery is no longer an option.

## Discussion

PDAC has an incredibly poor prognosis with a 5-year survival of less than 10%^45^. There has been little improvement in survival over the last 40 years and so new approaches are urgently required to improve patient outcome and decrease mortality. Surgical resection remains one of the only curative measures^46^; however, this is limited to 10–20% of patients with localized tumors. For the remaining patients, systemic chemotherapies remain the most common treatment option. However, it is generally accepted that in PDAC, the high levels of tumor desmoplasia play a crucial role in limiting the efficacy of current standard-of-care treatments^11^. As a result, an increasing number of studies are aimed at targeting this desmoplastic response to enhance efficacy of current chemotherapeutic regimens.

Preclinical studies targeting single members of the lysyl oxidase family (LOX and LOXL2) using antibody-based approaches have previously shown efficacy in in vivo models of cancer^16,19^, whereas others have shown negative outcomes^47^, suggesting both pro- and anti-tumorigenic roles for individual lysyl oxidase family member activity at different stages of tumor progression and metastasis (Extended Data Fig. 7c). The limited efficacy of studies to date is likely due to the combined involvement of all lysyl oxidase family members in disease progression.

Herein we introduce and characterize PXS-5505, a highly selective and potent pan-lysyl oxidase inhibitor with excellent oral pharmacokinetics. This small molecule is a pan-lysyl oxidase-targeting agent that has high selectivity for the entire lysyl oxidase family, while simultaneously showing no interaction with other human amine oxidases. PXS-5505 rapidly and irreversibly inhibits the lysyl oxidase family making it an excellent candidate as an antistromal therapy in highly desmoplastic tumors such as PDAC.

Our data show that PXS-5505 blocks lysyl oxidase family activity, chemotherapy-induced collagen crosslinking and increases in stiffness of the tumor microenvironment in human and mouse models.

PXS-5505 is well tolerated for 6 months in preclinical toxicity studies and >6 months in mouse PDAC models with no adverse side effects. In our KPC mouse and human PDX in vivo models of PDAC, PXS-5505 reduces collagen deposition and tumor stiffness and improves perfusion of agents into the primary tumor site. In the KPC model, PXS-5505 reduces spontaneous metastasis to visceral organs and most notably the liver, a major site of metastatic dissemination in patients. These in vivo findings match the in vitro organotypic data showing reductions in local invasion. Furthermore, addition of PXS-5505 to gemcitabine treatment extends median survival in the KPC model by approximately 45% and decreases metastatic deposits within the liver compared to gemcitabine alone.

Analysis of primary tumors shows that PXS-5505 decreases presence of highly fibrillar collagen, blunting the development of chemotherapy-induced desmoplasia, thereby improving tumor perfusion, decreasing tumor cell pSTAT3 activation and augmenting the anti-neoplastic effects of gemcitabine. It should be noted that a degree of desmoplasia is to be expected among all treatment groups as ongoing de novo synthesis of lysyl oxidase family members will enable a degree of collagen biosynthesis and tumor desmoplasia as a whole, is not exclusively composed of lysyl oxidase family crosslinked collagen.

In dissecting the effects of PXS-5505 on metastatic colonization of secondary organs, our data show that when used in combination with gemcitabine, PXS-5505 potentiates chemotherapy efficacy in both situations of early colonization and against late-stage established disease, the latter setting representing a large proportion of patients presenting in the clinic. Our data show that PXS-5505 has the potential to increase efficacy of gemcitabine against newly forming and established metastatic disease, demonstrating, in a model of established metastatic disease in pancreatic cancer, that a small-molecule pan-lysyl oxidase inhibitor can potentiate chemotherapy.

The oral pharmacokinetic profile of PXS-5505 shows fast absorption and clearance with a sufficiently long half-life to achieve efficient target engagement and inhibition of lysyl oxidase family activity. In our studies, we show that due to its superior drug-like properties compared to BAPN, PXS-5505 may also be dosed at concentrations five times lower (20 mg kg^−1^) than BAPN (100 mg kg^−1^) in animal studies^48^. Furthermore, PXS-5505 administration leads to rapid target inhibition in a variety of core and peripheral tissues and with lysyl oxidase activity recovery relying solely on de novo synthesis. Our preclinical data show no safety signals and no off-target activity on other amine oxidases, thus PXS-5505 is a strong candidate to be combined with chemotherapies in future clinical trials. Combining PXS-5505 with chemotherapies including gemcitabine as well as nab-paclitaxel or FOLFIRINOX, where tolerated, will be vital in determining efficacy in the clinic as these combination therapies have also been shown to induce desmoplasia in models of PDAC^49^.

In summary, PXS-5505 is a first-in-class small-molecule irreversible mechanistic pan-lysyl oxidase inhibitor that is well tolerated. Pharmacokinetic, pharmacodynamic, safety and antitumor activity data presented herein support a continuous daily dosing schedule for further clinical investigation. Inhibition of the lysyl oxidase family using a small-molecule inhibitor offers several benefits that target the tumor-associated stroma at several stages of cancer progression, including improved chemotherapy response, reduced metastatic burden and prolonged survival. Finally, our data present a compelling case for the continued transition into clinical trials as a stromal-targeting agent in combination with chemotherapy for the treatment of PDAC.

## Methods

### Animal ethics statement

All cancer models were used in strict accordance with the recommendations in the Australian Code of Practice for the Care and Use of Animals for Scientific Purposes by the National Health and Medical Research Council. The protocols (ARA 16/13, 19/06 and 19/08) were approved by the Garvan and St Vincent’s Precinct Animal Ethics Committee Genotyping of genetically engineered KPC mice was performed by the Garvan Molecular Genetics Facility (Sydney, Australia). All preclinical studies were approved by local ethics committees and, where applicable, following US Food and Drug Administration and Organization for Economic Cooperation and Development guidelines for Good Laboratory Practices in Association for Assessment and Accreditation of Laboratory Animal-accredited facilities.

### Patient ethics statements

Specimens of human pancreatic cancer CAFs were obtained through the HSA Biobank, UNSW Biorepository, UNSW Sydney, Australia from patients undergoing pancreatic resection and isolated following written informed consent (UNSW human ethics approval HC180973).

Ethics approval for acquisition and use of biological material for TMAs from the APGI and PDX material from the APMA (www.pancreaticcancer.net.au/APMA) was obtained from human research ethics committee (Sydney Local Health District Human Research Ethics Committee approval X16-0293).

### Cell culture

Primary KPC CCs and CAFs were isolated from KPC (*Pdx1-cre*; *LSL-Kras*^G12D/+^; *LSL-Trp53*^R172H/+^) tumors as described and used previously^28^. Specifically, CAFs were isolated according to the cell markers CD140a^+^/GP38^+^/EpCAM^−^/DAPI^−^. Validation was carried out by RNA-seq (Extended Data Fig. 2b), immunofluorescence staining (Extended Data Fig. 2a) and qPCR (Extended Data Fig. 2c). KPC CCs for in vivo implantation were engineered to express a luciferase bioluminescent-imaging biosensor using the pLV430G-oFL-T2A-eGFP construct^28,34^. KPC CCs and CAFs were routinely cultured in high-glucose DMEM (Gibco) supplemented with 10% FBS and 1% penicillin–streptomycin in a 21% O_2_/5% CO_2_ humidified incubator. The KPC CC and CAF identities and purity throughout our in vitro experiments were confirmed via morphological analysis and growth properties, qPCR with reverse transcription and immunofluorescence staining. Human-derived TKCC10 cells were maintained in 1:1 M199 media/Ham’s F12 medium (Gibco) supplemented with 7.5% FBS, 15 mM HEPES, 2 mM glutamine, 1× MEM vitamins, apotransferrin (25 ng ml^−1^), insulin (0.2 IU ml^−1^), 6.5 mM glucose, hydrocortisone (40 ng ml^−1^), EGF (20 ng ml^−1^), triiodothyronine (0.5 pg ml^−1^) and *O*-phosphoryl ethanolamine (2 μg ml^−1^) and cultured with 5% oxygen. Human CAFs were isolated from patients with PDAC tumors undergoing pancreatic resection. Explants were taken from histologically fibrotic areas of the pancreas and subsequent tissue blocks were cut and seeded onto uncoated culture wells and cultured at 37 °C in a 5% CO_2_ air-humidified atmosphere, cells grew out from the tissue blocks 1–3 d later and were used within 15 passages. CAFs were confirmed by immunohistochemistry for α-SMA, GFAP (positive) and cytokeratin (negative)^33,50^ and cultured in Iscove’s modified Dulbecco medium (IMDM), 10% FBS, 4 mmol l^−1^ l-glutamine in a 21% O_2_/5% CO_2_ humidified incubator. All cells were routinely confirmed as negative for the presence of *Mycoplasma*.

### Synthesis of PXS-5505

The three-step preparation of (*Z*)-3-fluoro-4-(quinolin-8-ylsulfonyl)but-2-en-1-amine dihydrochloride monohydrate (PXS-5505 dihydrochloride monohydrate) is detailed in Supplementary Fig. 1 (ref. ^20^). The resultant compound’s molecular weight was determined as described in Supplementary Figs. 2 and 3.

### Organotypic assays

High-purity rat tail collagen for 3D organotypic assays was acid extracted following previously published methodology^51^. The 12-day organotypic assays were carried out as described previously^52^. In brief, 2 × 10^5^ KPC CAFs were embedded in 2.5 ml 1.5 mg ml^−1^ rat tail collagen and allowed to remodel for 12 days in a six-well plate in DMEM supplemented with 10% FBS and 1% penicillin–streptomycin in 21% O_2_/5% CO_2_ (Extended Data Fig. 4a). For human pancreatic cancer CAFs, 3.45 × 10^5^ cells were embedded into 1.25 ml 1.5 mg ml^−1^ rat tail collagen and allowed to remodel for 12 days in a 12-well plate in IMDM supplemented with 4 mM glutamine and 10% FBS in 21% O_2_/5% CO_2_.

Following remodeling, 4 × 10^4^ KPC CCs were seeded on top of the remodeled matrix in DMEM supplemented with 10% FBS and 1% penicillin–streptomycin and allowed to grow to confluence for 4 days, after which matrices were transferred to an air–liquid interface and CCs allowed to invade into the organotypic matrices for 12 days (Extended Data Fig. 4f). PXS-5505 (30 μM) or PXS-5120 (100 nM) was added to medium at 48-h intervals for the duration. At the end point, matrices were fixed in 10% formalin and processed for histological analysis by H&E and panCK staining. The invasive index was measured in three representative areas per matrix and the number of invaded cells per mm^2^ was calculated. No statistical methods were used to predetermine sample sizes but our sample sizes are similar to those reported in previous publications^36,39^.

### Cell viability assays

The 2D cell viability assays were performed using KPC CAFs and CCs seeded at (250 CAFs per well) and (500 CCs per well) in a 96-well plate. MTS solution (Promega) was added to the cells at a 1:20 dilution and cells were incubated at 37 °C for 2 h. Cell proliferation rates were measured by detecting the absorbance at 490 nm using a microplate reader. Three biological repeats were performed.

The 3D cell viability assays were performed using cells embedded into polymerized collagen at 2.5 mg ml^−1^. KPC CAFs and CCs were seeded at 10,000 cells per well in a 96-well plate. MTS solution (Promega) was added to the cells at a 1:20 dilution and cells were incubated at 37 °C for 1.5 h. Cell proliferation rates were measured by detecting the absorbance at 490 nm using a microplate reader and three biological repeats were performed.

### KPC genetically engineered autochthonous model of PDAC

KPC (*Pdx1-cre*; *LSL-Kras*^G12D/+^; *LSL-Trp53*^R172H/+^) mice have been previously described^44^. Male and female mice were bred in-house on a mixed C57BL/6 background and kept in conventional caging with environmental enrichment, access to standard chow, water ad libitum and 12-h light–dark cycle at ambient temperature and humidity. Genotyping was performed in-house (Garvan Molecular Genetics Facility). Mice were monitored closely three times weekly and following detection of a palpable tumor (4–5 mm) (corresponding to late PanIN/early tumor development as detailed elsewhere^24,43,53^), which was verified by two independent researchers on two separate days, mice were randomized to receive vehicle (0.9% saline), gemcitabine (100 mg kg^−1^ i.p. twice weekly), PXS-5505 (20 mg kg^−1^ i.p.) or PXS-5505 (20 mg kg^−1^ i.p. daily) + gemcitabine (100 mg kg^−1^ i.p. twice weekly). The end point was determined once animals exhibited symptoms of advanced PDAC (including swollen abdomen, loss of body conditioning resembling cachexia, reduced mobility, development of ascites, overnight weight loss >10% of body weight, hunching posture and/or signs of distress and/or suffering, total weight loss ≥20% from maximum body weight or tumor interfering with mobility and access to food and water). The maximal tumor burden allowed by ethics was not exceeded. All mice where non-tumor-related complications led to a premature end point were censored.

### In vivo KPC PXS-5505 administration

For the KPC autochthonous model, gemcitabine HCl (Jomar Life Research) or vehicle control (0.9% saline) was administered by i.p. injection twice weekly (100 mg kg^−1^ in saline) on day 2 and 4 of the treatment cycle until the end point was reached. GLP-grade PXS-5505 (20 mg kg^−1^ in saline) or vehicle (0.9% saline) was administered daily by i.p. injection. Treatment of mice was initiated upon detection of a palpable tumor, verified by two independent researchers and monitoring of mice continued daily until the experimental end point. End points were defined as development of ascites, overnight weight loss >10% of body weight, hunching posture and/or signs of distress and/or suffering, total weight loss ≥20% from maximum body weight or tumor interfering with mobility and access to food and water. The maximal tumor burden allowed by ethics was not exceeded. At the end point, primary tumors and secondary metastatic organs (including liver, small intestine, diaphragm and lungs) were collected and samples were taken for formalin fixation overnight, unconfined compression analysis and snap-frozen for tissue banking. Fixed tissues were processed according to standard histology protocols and paraffin-embedded.

For the intrasplenic models, animals were randomized before receiving treatment. GLP-grade PXS-5505 (20 mg kg^−1^) was administered daily i.p. (starting day 0 for the early stage and day 4 for the late stage) until the end point and gemcitabine (100 mg kg^−1^) was administered on days 3, 6 and 9 for the early stage and days 5, 7 and 10 for the late stage.

### Intrasplenic injection of cancer cells

KPC CCs (5 × 10^5^ cells per mouse in 50 μl Hanks balanced saline solution) were slowly injected into the spleens of 8-week-old female BALB/c-Fox1nuAusb mice under anesthesia (isoflurane 3 l, O_2_ 1 l min^−1^ with vacuum) as previous described^34^. For the duration of the study mice were kept in conventional caging with environmental enrichment, access to standard chow, water ad libitum and 12-h light–dark cycle at ambient temperature and humidity. For intrasplenic studies, mice were culled at defined end points (10 d for early colonization studies and 11 d for late colonization studies). Animals with non-tumor-related complications that led to a premature end point were censored. At the end point, mice were imaged by whole-body bioluminescent imaging using an IVIS Spectrum (Caliper LS), culled and livers were removed for additional bioluminescent imaging before formalin fixing and paraffin embedding.

### Unconfined compression analysis: in vitro samples

Unconfined compression of 3D organotypic collagen matrices treated with or without PXS-5505 was performed on a TA Instruments Dynamic Hybrid Rheometer (DHR-3, TA Instruments). Remodeled collagen matrices were placed between upper and lower parallel plate geometries. A constant linear compressive rate of 5 µm s^−1^ was applied to the samples and axial force (N) and gap (mm) were collected every 0.01 s. Data were processed and a stress–strain curve for each replicate was obtained. Bulk elastic modulus (kPa) was obtained as the gradient of the linear region of the stress–strain curve corrected for cross-sectional area of sample and normalized to control. Data points represent biological replicates. No statistical methods were used to predetermine sample sizes but our sample sizes are similar to those reported in previous publications^34^.

### Unconfined compression analysis: in vivo samples

Unconfined compression of in vivo tissue samples was carried out on 2–3-mm cross-sectional slices using a TA Instruments Dynamic Hybrid Rheometer (DHR-3, TA Instruments). Tissue samples were placed between upper and lower parallel plate geometries. A constant linear compressive rate of 5 µm s^−1^ was applied to the samples and axial force (N) and gap (mm) were collected every 0.01 s. Data were processed and a stress–strain curve for each replicate was obtained. Bulk elastic modulus (kPa) was obtained as the gradient of the linear region of the stress–strain curve corrected for cross-sectional area of sample. Data points represent individual biological replicates.

### Subcutaneous PDX transplantation models (APGI and APMA)

Human PDAC specimens were obtained with human research ethics committee approval (X16-0293) from the APMA and APGI. PDX specimens were propagated and used under animal research ethics committee approval (ARA 16/13, ARA 19/06 and ARA 19/08).

Subcutaneous PDX studies were performed by implanting early passage PDX pieces (~4 mm^3^) or TKCC10 CCs (1.5 × 10^6^ cells per mouse in 100 μl PBS/Matrigel 1:1) into female (PDX) or male (TKCC10) 8-week-old immunocompromised nude Balb/c-Fox1nuAusb mice subcutaneously on the left flank. For the duration of the study, mice were kept in conventional caging with environmental enrichment, access to standard chow, water ad libitum and 12-h light–dark cycle at ambient temperature and humidity. Treatment began when tumors reached 150 mm^3^, where each mouse was randomized into a treatment group and administered gemcitabine HCl (100 mg kg^−1^ in saline) (Jomar Life Research) or vehicle control (0.9% saline) by i.p. injection twice weekly on day 2 and 4 of the treatment cycle until an ethical end point was reached. These included maximal tumor mass ≥10% of body weight, weight loss ≥20% from maximum body weight or tumor interfering with mobility and affecting access to food and water. Animals with non-tumor-related complications that led to a premature end point were censored. PXS-5505 (20 mg kg^−1^ in saline) or vehicle (0.9% saline) was administered daily by i.p. injection. Tumor size was monitored every second day by calipers. Tumor volume was calculated using the formula (length × width^2^)/2. At day 39, mice were killed via CO_2_ and cervical dislocation and tumors were collected and processed. The maximal tumor burden allowed by ethics was not exceeded.

### Western blot

Protein was extracted from 10 mg tissue lysed in RIPA buffer (150 mM NaCl, 1% Triton X-100, 0.05% sodium deoxycholate, 0.1% SDS and 50 mM Tris, pH 8.0) containing phosphatase inhibitor (1:1,000 dilution, sodium orthovanadate 0.1 M) and protease inhibitor cocktail (Roche). Samples were then spun at 13,300 r.p.m. at 4 °C for 10 min and normalized for loading by BCA assay (Pierce, 23225). Protein (15 µg) was loaded on a 10% Bis-Tris Gel using a mini-Bolt system (Life Technologies) and transferred using the mini Bolt system to a PVDF membrane (0.45-µm pore size, Merk) according to the manufacturer’s instructions. Primary LOX antibody (1:250 dilution) was incubated overnight followed by anti-rabbit secondary antibody (1:5,000 dilution) (Supplementary Table 3) for 1 h at room temperature and visualized using ECL Plus (Amersham, GE Healthcare). Validation for LOX antibody has been previously performed^15,16,54–56^. Ponceau staining for loading was performed by incubation for 15 min at room temperature and imaged on a flatbed scanner.

### H&E staining

All tissues or matrices were fixed in 10% buffered formalin and embedded in paraffin. The 4-μm sections of samples were deparaffinized with xylene and rehydrated in graded ethanol washes. H&E staining and counterstaining were performed on a Leica autostainer.

### Immunohistochemistry staining

All tissues or matrices, including patient TMAs, were fixed in 10% buffered formalin and embedded in paraffin. The 4-μm sections of samples were deparaffinized with xylene and rehydrated in graded ethanol washes. For immunohistochemistry, Bond Dewax Solution (AR9222) was used. Antigen retrieval was performed using Bond RX H2(30) protocol: HIER 30 min ER1 (citrate pH 6) OR ER2 (EDTA pH 9) at 100 °C. Slides were quenched in BOND Polymer Refine Detection (Leica Biosystems DS9800). Slides were incubated with primary antibodies (Supplementary Table 3) α-SMA (Abcam ab5694, 1:100 dilution), Ki67 (Thermo Fisher RM-9106-511, 1:500 dilution), pan-cytokeratin (Leica-Novostra C11, 1:50 dilution), PDGFR-β (Cell Signaling 3169 1:100 dilution), pMLC2 (Cell Signaling 3675S 1:100 dilution), CD31 (Taylor Bio-Medical DIA-310 1:100 dilution), pSTAT3 (Tyr705) (Cell Signaling 9131S 1:100 dilution), MPO (Agilent A039829-2 1:2,000 dilution), F4/80 (Abcam 100790 1:100 dilution), CD8 (Cell Signaling 98941,1:200 dilution) and CK19 (Abcam 133496) 1:1,000 dilution), followed by visualization by diaminobenzidine (DAB). H&E staining and counterstaining were performed on a Leica autostainer. Antibodies were validated by suppliers. Slides were digitized on an Aperio slide scanner and representative areas per tumor acquired and analyzed by researchers blinded to the conditions of the experiment.

### Analysis of lysyl oxidase family expression in patient datasets

All gene expression analysis was performed in R (v.3.6.1). Microarray data and clinicodemographic data from 269 patients with pancreatic cancer were downloaded from the APGI/ICGC data repository (https://dcc.icgc.org/). Microarray probes were mapped using the illuminaHumanv4.db and probes were collapsed to genes using the average probe value.

TCGA RNA-seq (RSEM normalized) and clinicodemographic information were downloaded using GDAC Firehose. The RNA-seq data were filtered for lowly expressed genes before being normalized using EdgeR.

The association of lysyl oxidase family gene expression with survival was assessed using Cox proportional hazards models (coxph package). A combined lysyl oxidase family score was generated by additively combining the expression of each lysyl oxidase family member weighted by its univariate Cox proportional hazards model coefficient. For visualization purposes, the continuous lysyl oxidase family scores were stratified by tertiles and Kaplan–Meier plots were generated using the survminer and survival packages. Patients were excluded from the analysis if the recorded cause of death was other than pancreatic cancer.

### Patient tissue microarrays from the APGI cohort

Biospecimens and clinical data were provided by the APGI (www.pancreaticcancer.net.au), which is supported by an Avner Pancreatic Cancer Foundation Grant (https://pankind.org.au/). Picrosirius red staining of the PDAC ICGC set arrays^21^ was performed on 4-μm sections that were deparaffinized and rehydrated before staining. Staining with 0.1% picrosirius red (Poly-sciences) was performed according to manufacturer’s instructions followed by counterstaining with hematoxylin. Dehydration and cover-slipping of slides was performed before simultaneous brightfield and polarized light tiled imaging on a Leica DM 6000 microscope fitted with posterior and anterior polarizing filters.

### Single-molecule array plasma testing

LOX and LOXL2 plasma concentrations were determined by Quanterix as previously described^22^, using commercially available antibodies (LOX, L4794; LOXL2, AF2639). Plasma samples from healthy individuals and patients with PDAC included both males and females. Plasma samples from patients with PDAC all came from patients with histologically confirmed PDAC, predominantly stages 3–4 (75% of samples). Healthy individuals had no clinical diagnosis of any disease.

### Quantification of liver metastases

The 4-µm formalin-fixed paraffin-embedded (FFPE) sections were cut and stained for H&E following standard histopathology methodologies or stained for CK19 using the Bond RX H2(30) protocol detailed above. Whole liver sections were then digitized on an Aperio slide scanner for blinded analysis. The total number of liver metastases were quantified and determined using QuPath^57^.

### Second harmonic generation imaging and analysis

SHG imaging of fibrillar collagens was employed to analyze collagen density and organization as performed previously^28,34,58^. In brief, SHG signal was acquired from 4-μm FFPE sections using a Leica DMI 6000 SP8 inverted multiphoton microscope with a 25 × 0.95 NA water objective. The excitation source was a Ti:Sapphire femtosecond laser cavity (Coherent Chameleon Ultra II) operating at 80 MHz and tuned at a wavelength of 880 nm. Intensity was recorded with a RLD HyD detector (440/20 nm). Three (organotypic matrices) or five (KPC tumors) 512 × 512 representative images were obtained per sample with a line average of 4 in a 3D stack (z-step 2.52 μm on cut tissue sections). SHG signal intensities were measured using MATLAB (MathWorks) as described previously^28,34,58^.

### Quantification of picrosirius red staining under transmitted and polarized light

Paraffin-embedded samples were cut into 4-μm sections, rehydrated and stained with 0.1% picrosirius red (Poly-sciences) for fibrillar collagen according to the manufacturer’s instructions. Slides were counterstained with hematoxylin, before dehydration and cover-slipping. Simultaneous brightfield and polarized light tiled imaging was performed on a Leica DM 6000 microscope fitted with posterior and anterior polarizing filters.

For assessment of picrosirius red staining in KPC and APMA patient tumors, five representative regions of interest per section were analyzed for both brightfield and polarized light by a blinded reviewer using in-house automated analysis scripts as described previously^28,34,58^ (see ‘Code availability’ statement). In tumors, regions of interest excluded areas of necrosis (where present) and tumor boundaries with healthy tissue.

To assess the association of picrosirius red staining with survival, the maximum picrosirius red signal (percent red-orange signal of total birefringent signal under polarizing light, equivalent to densely bundled, highly fibrillar collagen) was calculated across three cores per patient. This picrosirius red signal was combined with clinicodemographic information and the association with survival was assessed using Cox proportional hazards model (coxph package) in R (v.3.6.1). For visualization purposes, the continuous picrosirius red signals were stratified into quartiles and Kaplan–Meier plots were generated using the survminer survival packages.

To combine the lysyl oxidase expression scores with the picrosirius red score, the lysyl oxidase family expression scores were first stratified into tertiles valued at 1, 2 and 3. Similarly, the continuous birefringent percent red-orange signal was stratified into quartiles valued at 1, 2, 3 and 4. The combined scores were multiplicative combinations of the lysyl oxidase family score with the birefringent percent red-orange signal. This continuous variable was then quartiled and survival was assessed using the Cox proportional hazards model (coxph package) and visualized using Kaplan–Meier plots as described above.

### Whole-body IVIS spectrum imaging

The luciferase signal was imaged to monitor progression of intrasplenic tumors on a Caliper Life Sciences IVIS spectrum. Luciferin (150 mg kg^−1^, Gold Biotechnology) was administered by i.p. injection. Imaging was performed on mice while under anesthesia (isoflurane 3 l, in O_2_ 1 l min^−1^ with vacuum). The signal was acquired with open filters and small binning. Radiance was used as a measure of signal intensity to confirm tumor presence.

### FITC-dextran imaging

FITC-dextran (10 kDa) was administered at 40 mg ml^−1^ in saline via tail vain 2 h before culling. Excised tumors were flash-frozen in OCT and cryosectioned at 14 μm. Sections were counterstained with 4,6-diamidino-2-phenylindole (DAPI) and images were collected using a Leica DMI5500 (×40 magnification). Images were quantified using Fiji Software for Integrated Density Signal.

### Jump dilution assay

To measure recovery of activity, the enzyme (either human recombinant hrLOXL1 (used as a surrogate for LOX owing to the similar pharmacology) or hrLOXL2) was incubated with 10 × IC_50_ of the inhibitor (PXS-5505) for 30 min then diluted 100-fold. A standard Amplex red assay was completed to determine the level of recovery of enzyme activity after the dilution.

### Amplex red enzymatic activity assay on cell-derived conditioned medium

Cell-derived conditioned medium was prepared from KPC CAFs and CCs grown in phenol red-free DMEM with 0.1% FBS and 10 μM CuSO_4_ for 24 h. At collection, size filtration >10 kDa for 40× concentration and buffer exchange (1.2 M urea and 50 mM sodium borate buffer, pH 8.2) were performed and subsequently used for the Amplex red assay as previously described^59^ using putrescine as a substrate. Three biological repeats were performed.

### Real-time PCR

A total of 5 × 10^5^ KPC CCs or CAFs were seeded and collected after incubation in 21% O_2_/5% CO_2_ for 72 h. RNA was isolated using the RNeasy mini kit (QIAGEN) according to the manufacturer’s instructions. Then, 1 μg of RNA was reverse transcribed using the Transcriptor First Strand cDNA Synthesis kit (Roche LifeScience) with anchored-oligo(dT)_18_ primers or the QuantiTect Reverse Transcription kit (QIAGEN). Real-time PCR was performed using the Roche LightCycler480 (Roche LifeScience) or QuantStudio 7 (Thermo Fisher). The following probe sets from Applied Biosystems, were used: *LOX*, Mm00495386; *LOXL1*, Mm01145738; *LOXL2*, Mm00804740; *LOXL3*, Mm01184867; *LOXL4*, Mm00446385; and *GAPDH*, Mm99999915. Primers and Roche Universal Probe Library probes used for RT–qPCR are described in Supplementary Table 4. Relative mRNA expression levels for each transcript of interest were normalized to *GAPDH* or *ACTB* (as indicated) and quantified using the comparative double Δ*C*_t_ method (unless stated otherwise) for each biological replicate.

### Immunofluorescence staining and imaging

A total of 1 × 10^4^ CCs or CAFs were seeded onto 12-mm glass coverslips (ProSciTech, *n* = 3 per cell line) and incubated in 21% O_2_/5% CO_2_. After 72 h, cells were washed with DPBS and fixed in 4% PFA for 10 min at room temperature. Cells were then permeabilized with ice-cold methanol for 10 min, blocked with 2.5% BSA, 5% normal donkey serum and 0.02% glycine for 1 h at room temperature and incubated with primary antibody (CDH1 1:200 dilution, BD Biosciences 610181; α-SMA 1:200 dilution, Abcam ab5694) overnight at 4 °C. Cells were incubated with the Cy3 AffiniPure F(ab′)_2_ fragment donkey anti-mouse secondary antibody (1:500 dilution, Jackson ImmunoResearch Laboratories (CDH1)) or Cy3 AffiniPure F(ab′)_2_ fragment donkey anti-rabbit secondary antibody (1:500 dilution, Jackson ImmunoResearch Laboratories (α-SMA)) (Supplementary Table 3) for 1 h at room temperature, counterstained with 4,6-diamidino-2-phenylindole (DAPI) and mounted in ProLong Diamond Antifade Mountant (Thermo Fisher). Stained cells were then imaged using a DMI 6000 SP8 confocal microscope (Leica).

### RNA-seq

RNA was extracted from KPC CAFs (in triplicate) using the QIAGEN AllPrep kit. RNA concentration and integrity were assessed using the Agilent 4200 Tapestation system, which confirmed sample RINs >9. Library preparation was performed using the KAPA mRNA HyperPrep kit according to manufacturer’s protocol (Roche) and paired-end sequencing was performed using the Illumina NovaSeq 6000. The 150-bp paired-end reads were processed using Trim Galore (v.0.4.0) for adaptor trimming and STAR (v.2.4.0d) for mapping reads to the mm10/GRCm38 mouse genome build, with GENCODE v.M13 used as a reference transcriptome. Mapped reads were counted into genes using rsem (v.1.2.21)^60^.

### Mass spectrometry crosslink analysis

Collagen matrices were freeze dried and reduced with NaBH_4_. The resulting pellet was hydrolyzed with 6 mol l^−1^ HCl at 100 °C for 24 h. An automated solid phase extraction system (Gilson GX-271 ASPECA system) was used to extract hydroxyproline and crosslinks from the hydrolysate. The samples were analyzed for hydroxyproline crosslinks by a UHPLC–ESI–MS/MS on a Thermo Dionex UPHPLC and TSQ Endura triple quadmass spectrometer as previously published^61^. The lower limit of detection was 0.013 pmol 10 µl^−1^.

### Statistics and reproducibility

Details of individual data can be found in the corresponding figure legends. For in vitro data, biological triplicates were performed unless otherwise stated and in vivo, ten animals per group were enrolled unless otherwise stated. Data distribution was assumed to be normal, but this was not formally tested. Unpaired two-group comparisons were performed using the Mann–Whitney *U*-test, unpaired multi-group comparisons by one-way analysis of variance and Kaplan–Meier curves were compared using the Mantel–Cox log-rank test. Where multiple tests were performed, the familywise error rate was controlled by the Holm-Sidak step-up method. GraphPad Prism v.8 and 9 or R (v.3.6.1) were used for all analyses.

### Reporting summary

Further information on research design is available in the Nature Portfolio Reporting Summary linked to this article.

### Supplementary information

Supplementary Information Supplementary Figs. 1–3

Reporting Summary

Supplementary Table Supplementary Tables 1–4 (separate tabs).

### Source data

Source Data Figs. 1–5 and Extended Data Figs. 1–7 Statistical Source Data.

Source Data Extended Data Fig. 1 Unprocessed western blot. **a**, Primary LOX antibody (1:250 dilution) visualized using ECL Plus (Amersham, GE Healthcare) **b**, Ponceau stain.

## Data Availability

The data that support the findings of this study are available from the corresponding author upon request. The human PDAC data were derived from the TCGA Research Network at http://cancergenome.nih.gov/. Structural information of LOXL2 used in Extended Data Fig. 1 was derived from PDB ID: 5ZE3. Materials and data from the APGI and APMA can be provided by the APGI and APMA pending scientific review and a completed material transfer agreement. The RNA-seq data have been deposited and can be accessed via the accession code GSE186748. All other data supporting the findings of this study are available from the corresponding author on reasonable request. Source data are provided with this paper.

## Code availability

ImageJ/FIJI scripts for the automated analysis of images are freely available through GitHub (www.github.com/tcox-lab).
