# Supplementary material for: A first-in-class pan-lysyl oxidase inhibitor impairs stromal remodeling and enhances gemcitabine response and survival in pancreatic cancer
Source: Nat Cancer. 2023 Aug 28;4(9):1326–44. doi: 10.1038/s43018-023-00614-y (PMC10518255; doi:10.1038/s43018-023-00614-y)
Supplement: Supplementary file 1 — Supplementary Figs. 1–3 [file 43018_2023_614_MOESM1_ESM.pdf]

nature cancer

Article

https://doi.org/10.1038/s43018-023-00614-y

A first-in-class pan-lysyl oxidase inhibitor impairs stromal remodeling and enhances gemcitabine response and survival in pancreatic cancer

In the format provided by the authors and unedited

Supplementary data 1: Preparation of (*Z*)-3-fluoro-4-(quinolin-8-ylsulfonyl)but-2-en-1-amine dihydrochloride monohydrate (PXS-5505 dihydrochloride monohydrate)

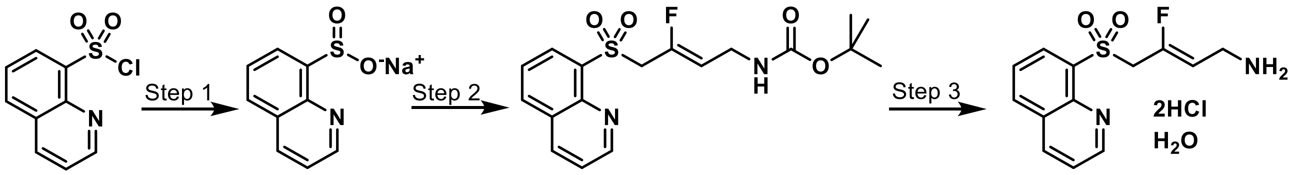

Step 1: Preparation of sodium quinoline-8-sulfinate

A vessel charged with Na_2SO_3 (6.70 kg, 53.2 mol) and water (21.0 L) was stirred at room temperature for 20 min. To the vessel was added Na_2CO_3 (5.50 kg, 51.9 mol) and stirring was continued at room temperature for 20 min. Quinoline-8-sulfonyl chloride (6.00 kg, 26.4 mol) was then added portionwise while maintaining the temperature below 40 °C. The resulting mixture was stirred at room temperature for 3 h. The reaction mixture was filtered and the filter cake was washed with methanol (7.0 L). The filtrate was concentrated to dryness *in vacuo*, and to the resulting residue was added methanol (7.0 L). After stirring at room temperature for 1 h, the mixture was again filtered and the filtrate concentrated to dryness. In a second, and final, washing cycle the residue was taken up in methanol (10.0 L) and stirring was continued at room temperature for 1 h. The mixture was filtered and the filtrate was concentrated *in vacuo* to afford sodium quinoline-8-sulfinate (4.10 kg) that was used in the next step.

Step 2: Preparation of *tert*-butyl (*Z*)-(3-fluoro-4-(quinolin-8-ylsulfonyl)but-2-en-1- yl)carbamate

A vessel charged with *tert*-butyl (*Z*)-(4-bromo-3-fluorobut-2-en-1-yl)carbamate (prepared according to reference[1]; 3.50 kg, 13.1 mol), sodium quinoline-8-sulfinate (4.20 kg, 19.5 mol), prepared as described in Step 1, and *N*,*N*-dimethylformamide (17.5 L) was cooled to 15 – 20 °C. The resulting mixture was stirred at this temperature for 20 h. The mixture was then diluted with ethyl acetate (35.0 L) and water (35.0 L), and stirring was continued for a further 10 min. The organic layer was then separated and washed with water (20.0 L x 2). After concentrating the organic layer to approximately 20 L, *n*-heptane (42.0 L) was added drop-wise. The resulting

suspension was stirred at 20 – 30 °C for 20 h. The solid was isolated by filtration, washed with *n*-heptane and then dried under vacuum at 50 – 55 °C for 20 h to afford *tert*-butyl (*Z*)-(3-fluoro-4- (quinolin-8-ylsulfonyl)but-2-en-1-yl)carbamate (3.80 kg, 77%). Retention time = 12.97 min; Method - Agilent LC/MSD 1200 Series; column: ZORBAX SB-C18, ODS 2000 (50 × 4.6 mm, 5 µm) operating in ES (+) or (-) ionization mode; flow rate 1.5 mL/min; Temperature (T) = 30 °C; detection wavelength: 214 nm; mobile phase: from 5% acetonitrile (containing 0.05% trifluoroacetic acid (TFA)) and 95% water (containing 0.05% TFA) to 90% acetonitrile and 10% water, over 24 min.

Step 3: Preparation of (*Z*)-3-fluoro-4-(quinolin-8-ylsulfonyl)but-2-en-1-amine dihydrochloride monohydrate (PXS-5505 dihydrochloride monohydrate)

To a vessel charged with HCl (1.5 M in ethyl acetate; 53 L) at 10 – 20 °C was added *tert*-butyl (*Z*)-(3-fluoro-4-(quinolin-8-ylsulfonyl)but-2-en-1-yl)carbamate (5.3 kg, 14 mol) prepared as described in Step 2. The mixture was stirred at 15 °C for 4 h. The resulting solid was isolated by filtration and washed with ethyl acetate (20 L). To a flask containing the solid was added ethyl acetate (53 L). The suspension was then stirred at 10 – 20 °C for 2 h. The solid was again isolated by filtration and washed with ethyl acetate (20 L). The solid was dissolved in methanol (53 L) and the solution was filtered. To this was then added water (500 mL) and *tert*-butyl methyl ether (53 L) drop-wise and stirring was continued at 15 °C for a further 20 h. The solid was collected by filtration and dried under vacuum at 55 – 60 °C to afford (*Z*)-3-fluoro-4-(quinolin-8-ylsulfonyl)but-2-en-1-amine dihydrochloride monohydrate (PXS-5505 dihydrochloride monohydrate) (4.4 kg, 90%). ^{1}H NMR (400 MHz, D2O) δ ppm: 8.96 (dd, *J* = 5.4, 1.8 Hz, 1H), 8.65 (dd, *J* = 8.6, 1.4 Hz, 1H), 8.34 (dd, *J* = 7.6, 0.8 Hz, 1H), 8.24 (dd, *J* = 8.2, 1.0 Hz, 1H), 7.77 (dd, *J* = 8.6, 5.0 Hz, 1H), 7.71 (t, *J* = 7.8 Hz, 1H), 5.02 (dt, *J* = 33.6, 7.3 Hz, 1H), 4.61 (d, *J* = 19.2 Hz, 2H), 3.47 (d, *J* = 7.2 Hz, 2H); LCMS: for C13H13FN2O2S calculated 280.1, found 281.1 $[M+H]^{+}$.

Supplementary data 2: ^{1}H NMR spectrum of PXS-5505

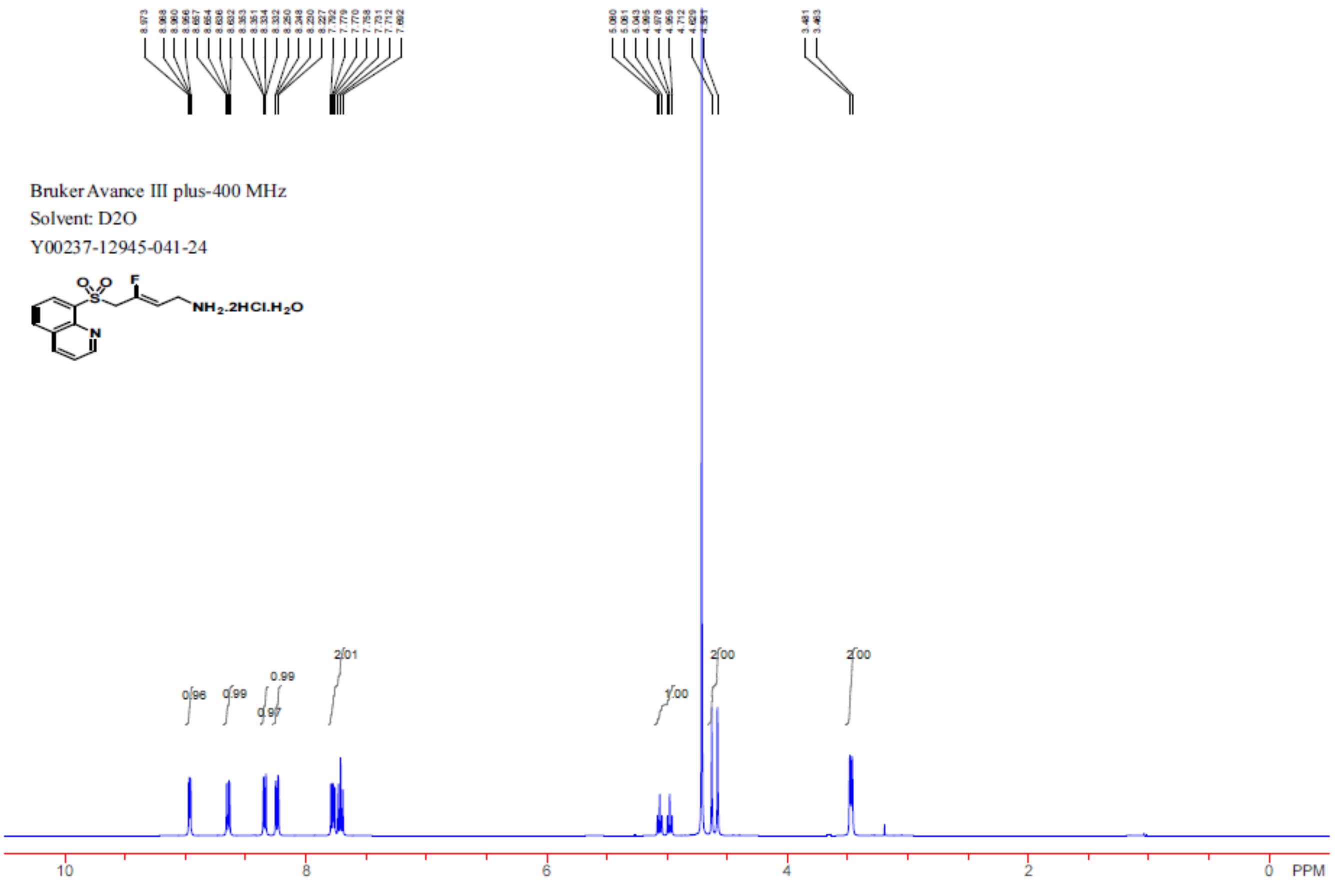

Supplementary data 3: Liquid chromatography–mass spectrometry (LCMS) data for PXS-5505

Method: Data was recorded on a Shimadzu LC-MS 2020 instrument operating in ES (+) or (-) ionization mode; LC-MS column Restek Raptor ARC-18 5um (4.6x150mm); mobile phase A: 0.1% formic acid in Milli Q Water, mobile phase B: 0.1% formic acid in methanol (gradient grade); flow rate: 1.5 mL/min; column oven temperature: 40 °C; run time: 9.4 min; injection volume: 3 μL; binary gradient flow program: 95% water (0.1% formic acid) and 5% methanol (0.1% formic acid) to 50% water (0.1% formic acid) and 50% methanol (0.1 % formic acid) over 1.40 min; then to 5% water (0.1% formic acid) and 95% methanol (0.1 % formic acid) at 6.00 min; holding the condition until 7.40 min, bringing back to 95% water (0.1% formic acid) and 5% methanol (0.1 % formic acid) from 7.41 min to 9.40 min.

a) LC trace (254 nm):

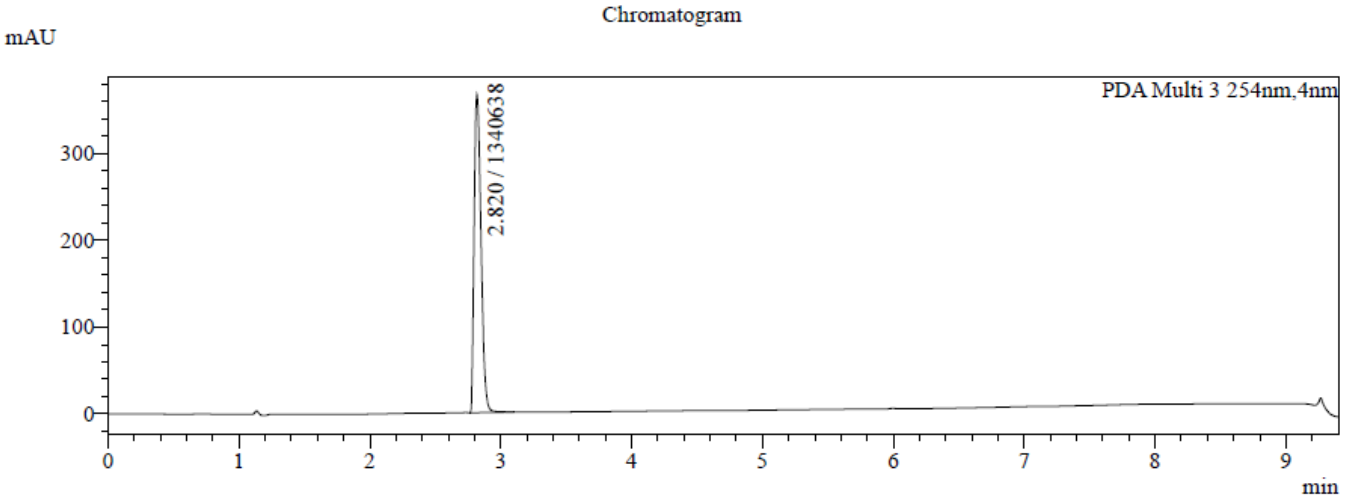

Peak Table

PDA Ch3 254nm

Peak#	Ret. Time	Area	Area%
1	2.820	1340638	100.000
Total		1340638	100.000

b) MS of the product peak (ES(+) ionization mode):

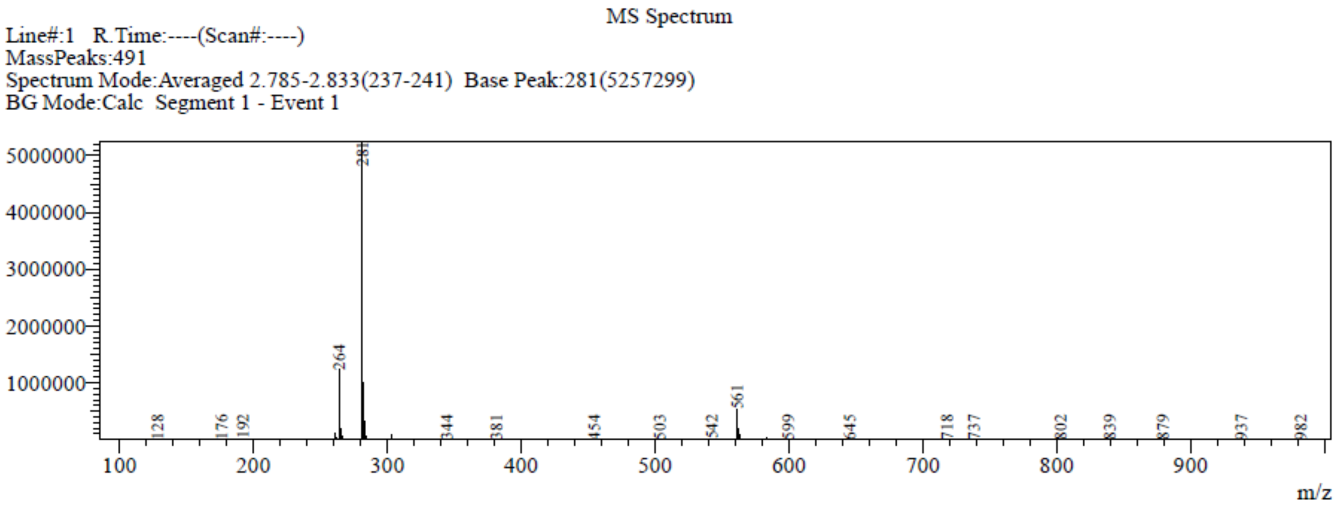

Reference:

1. Findlay, A. D., Foot, J. S., Buson, A., Deodhar, M., Jarnicki, A. G., Hansbro, P. M., Liu, G., Schilter, H., Turner, C. I., Zhou, W. & Jarolimek, W. Identification and Optimization of Mechanism-Based Fluoroallylamine Inhibitors of Lysyl Oxidase-Like 2/3. *J. Med. Chem.* 62, 9874-9889 (2019).
