# Supplementary material for: A first-in-class pan-lysyl oxidase inhibitor impairs stromal remodeling and enhances gemcitabine response and survival in pancreatic cancer
Source: Nat Cancer. 2023 Aug 28;4(9):1326–44. doi: 10.1038/s43018-023-00614-y (PMC10518255; doi:10.1038/s43018-023-00614-y)
Supplement: Supplementary file 2 — Reporting Summary [file 43018_2023_614_MOESM2_ESM.pdf]

nature portfolio

Corresponding author(s): Thomas R. Cox
Wolfgang Jarolimek

Last updated by author(s): Jun 22, 2023

Reporting Summary

Nature Portfolio wishes to improve the reproducibility of the work that we publish. This form provides structure for consistency and transparency in reporting. For further information on Nature Portfolio policies, see our Editorial Policies and the Editorial Policy Checklist.

Statistics

For all statistical analyses, confirm that the following items are present in the figure legend, table legend, main text, or Methods section.

n/a	Confirmed	
☐	☒	The exact sample size (*n*) for each experimental group/condition, given as a discrete number and unit of measurement
☐	☒	A statement on whether measurements were taken from distinct samples or whether the same sample was measured repeatedly
☐	☒	The statistical test(s) used AND whether they are one- or two-sided<br>*Only common tests should be described solely by name; describe more complex techniques in the Methods section.*
☐	☒	A description of all covariates tested
☐	☒	A description of any assumptions or corrections, such as tests of normality and adjustment for multiple comparisons
☐	☒	A full description of the statistical parameters including central tendency (e.g. means) or other basic estimates (e.g. regression coefficient) AND variation (e.g. standard deviation) or associated estimates of uncertainty (e.g. confidence intervals)
☐	☒	For null hypothesis testing, the test statistic (e.g. *F*, *t*, *r*) with confidence intervals, effect sizes, degrees of freedom and *P* value noted<br>*Give P values as exact values whenever suitable.*
☒	☐	For Bayesian analysis, information on the choice of priors and Markov chain Monte Carlo settings
☒	☐	For hierarchical and complex designs, identification of the appropriate level for tests and full reporting of outcomes
☒	☐	Estimates of effect sizes (e.g. Cohen's *d*, Pearson's *r*), indicating how they were calculated

Our web collection on statistics for biologists contains articles on many of the points above.

Software and code

Policy information about availability of computer code

Data collection	Second Harmonic Generation Imaging was undertaken using Leica DMI 6000 SP8 inverted multiphoton microscope<br>Histological imaging was undertaken using Leica DM 6000 microscope<br>IVIS imaging was undertake on a Caliper Life Sciences IVIS spectrum<br>Unconfined compression analysis bulk modulus data was collected using a DHR3 Dynamic Hybrid Rheometer (TA Instruments) using TRIOS Data acquisition software V5.1.1 (TA Instruments)<br>Quantitative real-time PCR was performed using the Roche LightCycler480 (Roche LifeScience) or QuantStudio 7 (Thermo Fisher)<br>Paired-end sequencing was performed using the Illumina NovaSeq 6000.<br>Confocal imaging was undertaken using a Leica DMI5500<br>Mass spectrometery was undertaken using Thermo Dionex UPHPLC and TSQ Endura triple quadmass spectrometer
Data analysis	Data analysis was carried out using Prism V8 & 9, R version 3.6.1, and MATLAB R2020<br>In house ImageJ scripts are available via GitHub (https://github.com/TCox-Lab) or from corresponding authors.<br>The RNAseq data was normalised using EdgeR.<br>For RNAseq, 150 bp paired-end reads were processed using Trim Galore (version 0.4.0) for adapter trimming and STAR (version 2.4.0d) for mapping reads to the mm10/GRCm38 mouse genome build, with GENCODE vM13 used as a reference transcriptome. Mapped reads were counted into genes using rsem (version 1.2.21)

For manuscripts utilizing custom algorithms or software that are central to the research but not yet described in published literature, software must be made available to editors and reviewers. We strongly encourage code deposition in a community repository (e.g. GitHub). See the Nature Portfolio guidelines for submitting code & software for further information.

Data

Policy information about availability of data

All manuscripts must include a data availability statement. This statement should provide the following information, where applicable:

- Accession codes, unique identifiers, or web links for publicly available datasets
- A description of any restrictions on data availability
- For clinical datasets or third party data, please ensure that the statement adheres to our policy

The data that support the findings of this study are available from the corresponding author upon request. The human PDAC data were derived from the TCGA Research Network: http://cancergenome.nih.gov/. Structural information of LOXL2 used in extended data figure 1 was derived from PDB ID: 5ZE3. Materials and data from APGI and APMA can be provided by APGI and APMA pending scientific review and a completed MTA. The RNA-seq data has been deposited and can be accessed via the accession code: GSE186748. Further information on research design is available in the Nature Research Reporting Summary linked to this article.

Research involving human participants, their data, or biological material

Policy information about studies with human participants or human data. See also policy information about sex, gender (identity/presentation), and sexual orientation and race, ethnicity and racism.

Reporting on sex and gender	Biobanked human plasma samples were purchased from a commercially established biobank (BioIVT) that fully anonymised samples. Reporting on sex and gender was not disclosed to researchers at the time of request. Samples were chosen by BioIVT based on availability from their biobank.
Reporting on race, ethnicity, or other socially relevant groupings	Biobanked plasma samples were purchased from a commercially established biobank (BioIVT) that fully anonymised samples. Reporting on race, ethnicity, or other socially relevant groupings was not disclosed to researchers at the time of request. Samples were chosen by BioIVT based on availability from their biobank.
Population characteristics	Biobanked plasma samples were purchased from a commercially established biobank (BioIVT) that fully anonymised samples. Reporting on Population characteristics was not disclosed to researchers at the time of request. Samples were chosen by BioIVT based on availability from their biobank.
Recruitment	This was a retrospective study and no recruitment was specifically undertaken for this study.
Ethics oversight	BioIVT have IRB approval from the FDA, ethics protocol numbers: 5035, 15002, 800962.

Note that full information on the approval of the study protocol must also be provided in the manuscript.

Field-specific reporting

Please select the one below that is the best fit for your research. If you are not sure, read the appropriate sections before making your selection.

☒ Life sciences ☐ Behavioural & social sciences ☐ Ecological, evolutionary & environmental sciences

For a reference copy of the document with all sections, see nature.com/documents/nr-reporting-summary-flat.pdf

Life sciences study design

All studies must disclose on these points even when the disclosure is negative.

Sample size	For animal experiments, groups of 20 or more animals randomised to each group, were used provide sufficient power to detect a 20% difference in survival with 99% confidence and a significance of 0.05. Statistical methods were not used to predetermine sample size in other in vitro experiments.
Data exclusions	All mice where non-tumour related complications led to a premature endpoint (as determined by animal ethics) were censored as detailed in the methodology. Maximal tumour burden allowed by ethics was not exceeded.
Replication	In vivo experiments were conducted with n=10 animals per group unless otherwise stated. The number of times experiments were independently repeated is indicated in the figure legends. All attempts to replicate the data were successful.
Randomization	Animals were randomised prior to enrollment into treatment groups. For non-animal (in vitro) experiments, the experimental design meant that randomisation was not applicable.
Blinding	Prior to enrollment, tumour presence was verified by two independent researchers on two separate days. Once confirmed, animals were randomised to a treatment group, however researchers were not blinded to the treatment being administered due to WH&S requirements in correct labeling and disposal of cytotoxic materials. Blinding was not possible for in vitro experiments as each independent experiment was carried out by an individual investigator that were involved in both data collection and experimental design. Where possible automated scripts were used for image analysis to minimise bias.

Reporting for specific materials, systems and methods

We require information from authors about some types of materials, experimental systems and methods used in many studies. Here, indicate whether each material, system or method listed is relevant to your study. If you are not sure if a list item applies to your research, read the appropriate section before selecting a response.

Materials & experimental systems

n/a	Involved in the study
☐	☒ Antibodies
☐	☒ Eukaryotic cell lines
☒	☐ Palaeontology and archaeology
☐	☒ Animals and other organisms
☒	☐ Clinical data
☒	☐ Dual use research of concern
☒	☐ Plants

Methods

n/a	Involved in the study
☒	☐ ChIP-seq
☒	☐ Flow cytometry
☒	☐ MRI-based neuroimaging

Antibodies

Antibodies used

A list of all antibodies used is provided in extended data table 4 and below:
LOX, Rabbit Polyclonal, Open Biosystems 1:250 WB
LOX, L4794, Rabbit Polyclonal,Sigma Aldrich SiMoA
LOXL2, AF2639 ,Goat Polyclonal, R&D systems SiMoA
alphaSMA, ab5694, Rabbit Polyclonal, Abcam 1:100 IHC, 1:200 IF
Ki67, RM9106S, Rabbit Monoclonal, Thermo Scientific 1:500 IHC
CD31, DIA-310, Rat Monoclonal, Dianova 1:100 IHC
MPO, A039829-2, Rabbit Polyclonal, Agilent 1:2000 IHC
F4/80, 100790, Rabbit Polyclonal, Abcam 1:100 IHC
CD8, 98941, Rabbit Monoclonal, Cell Signalling Technologies 1:200 IHC
PDGFR-beta, 3169, Rabbit Monoclonal, Cell Signalling Technologies 1:100 IHC
pMLC2, 3675S, Mouse Monoclonal, Cell Signalling Technologies 1:100 IHC
pSTAT3, 9131S, Rabbit Polyclonal, Cell Signalling Technologies 1:100 IHC
Pan-cytokeratin, 4568101318, Mouse Monoclonal, Leica Norostra 1:50 IHC
CDH1, 610181, Mouse Monoclonal, BD Bioscience 1:200 IF
CK19, 133496, Abcam 1:1000 IHC
Amersham ECL Rabbit IgG HRP-linked, NA934, Donkey Polyclonal, GE Healthcare Life Sciences 1:5000 WB
Cy™3 AffiniPure F(ab')$_2$ Fragment Donkey Anti-Mouse, 715-166-151, Donkey Polyclonal, Jackson ImmunoResearch Laboratories Inc 1:500 IF
Cy™3 AffiniPure F(ab')$_2$ Fragment Donkey Anti-Rabbit, 711-166-152, Donkey Polyclonal, Jackson ImmunoResearch Laboratories Inc 1:500 IF

Validation

LOX - Validated in "Erler et al. Cancer Cell (2009); Baker et al. JNCI (2011); Baker et al, Cancer Research (2013); Baker et al, Oncogene (2013), Cox et al Cancer Research (2013); Miller et al. EMBO Mol. Med. (2015)"
aSMA - Supplier validated - "Valid for 12 months from date of delivery"
Ki67 - Supplier validated - "Keszthelyi R et al. Immunomorphological Assessment of the New Ki-67 Specific Rabbit Monoclonal SP6 Antibody on Fixed-embedded Tissue Sections. Submitted for publication in Pathology Oncology Research"
F4/80 - Supplier validated - "Valid for 12 months from date of delivery"
Pan-cytokeratin - Supplier validated - "The performance of NCL-L-C11 has been validated on a range of normal and abnormal tissues"
No other specific validation statements were provided for the other antibodies.

Eukaryotic cell lines

Policy information about cell lines and Sex and Gender in Research

Cell line source(s)

Primary KPC cancer cells (CCs) and cancer associated fibroblasts (CAFs) were isolated from KPC (Pdx1-Cre; LSL-KrasG12D/+; LSL-Trp53R172H/+) tumours as described and used in (Vennin et al. 2019)

Specimens of human pancreatic cancer CAFs were obtained from the HSA Biobank, UNSW Biorepository, UNSW Sydney, Australia from patients undergoing pancreatic resection and isolated (as detailed in methods above) following written informed consent (UNSW human ethics approval # HC180973).

Authentication

No authentication of primary derived cancer cell lines from KPC mouse model undertaken, however validation of CAF and cancer cell markers was carried out by RNAseq, and immunofluorescence staining and/or qPCR

Mycoplasma contamination

Cells were routinely tested and confirmed negative for mycoplasma.

Commonly misidentified lines
(See ICLAC register)

No such cell lines were used.

Animals and other research organisms

Policy information about studies involving animals; ARRIVE guidelines recommended for reporting animal research, and Sex and Gender in Research

Laboratory animals	KPC (Pdx1-Cre; LSL-KrasG12D/+; LSL-Trp53R172H/+) male and female 10-12 weeks of age at enrollment<br>BALB/c-Fox1nuAusb female or male mice aged 8 weeks at enrollment
Wild animals	The study did not involve wild animals
Reporting on sex	Both male and female animals were used in the studies
Field-collected samples	The study did not involve samples collected from the field.
Ethics oversight	Protocols 16/13, 19/06 and 19/08 were approved by the Garvan and St Vincent's Precinct Animal Ethics Committee (AEC).

Note that full information on the approval of the study protocol must also be provided in the manuscript.
