# Supplementary material for: A first-in-class pan-lysyl oxidase inhibitor impairs stromal remodeling and enhances gemcitabine response and survival in pancreatic cancer
Source: Nat Cancer. 2023 Aug 28;4(9):1326–44. doi: 10.1038/s43018-023-00614-y (PMC10518255; doi:10.1038/s43018-023-00614-y)

Unmodified western blot from Extended data figure 1K

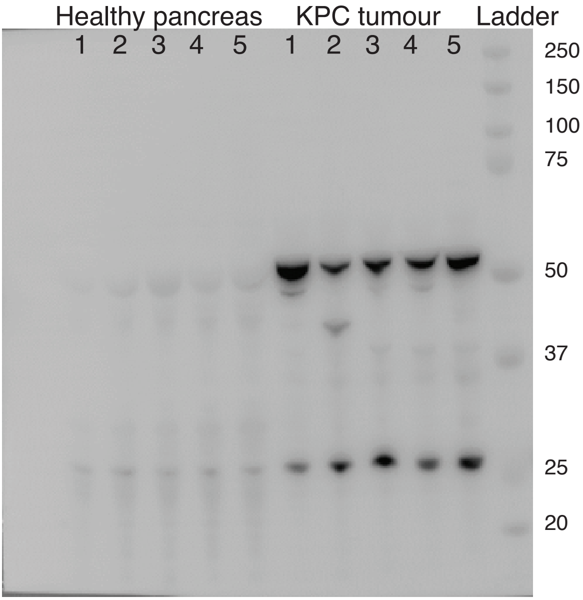

Ponceau staining of western blot from Extended data figure 1K

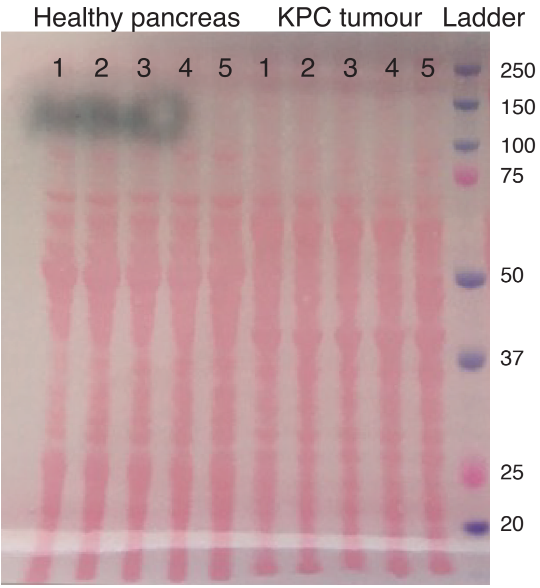

Supplement: Supplementary file 5 — Unprocessed western blot. a, Primary LOX antibody (1:250 dilution) visualized using ECL Plus (Amersham, GE Healthcare) b, Ponceau stain. [file 43018_2023_614_MOESM5_ESM.pdf]
